# APTES-functionalized Gd_0.18_Fe_2.82_O_4_@SiO_2_ nanocarrier for magnetothermal-triggered doxorubicin release

**DOI:** 10.1039/d6ra00215c

**Published:** 2026-03-11

**Authors:** Pham Hoai Linh, Tran Thi Huong, Nguyen Hong Nhung, Tran Thi Ngoc Nha, Pham Thanh Phong

**Affiliations:** a Institute of Materials Science, Vietnam Academy of Science and Technology 18-Hoang Quoc Viet Hanoi City Vietnam; b Graduate University of Science and Technology, Vietnam Academy of Science and Technology 18-Hoang Quoc Viet Hanoi City Vietnam; c Laboratory of Magnetism and Magnetic Materials, Science and Technology Advanced Institute, Van Lang University Ho Chi Minh City Vietnam phamthanhphong@vlu.edu.vn; d Faculty of Applied Technology, School of Technology, Van Lang University Ho Chi Minh City Vietnam

## Abstract

Externally regulated and stimuli-responsive drug delivery systems remain of significant interest for improving the controllability of cancer treatment strategies. In this study, APTES-functionalized Gd_0.18_Fe_2.82_O_4_@SiO_2_ core–shell nanoparticles were developed as a pH- and magnetically responsive platform for doxorubicin (DOX) delivery. Structural and morphological characterization confirmed quasi-spherical nanoparticles with mesoporous silica shells and satisfactory colloidal stability under physiological conditions. FTIR analysis indicated successful DOX adsorption mediated by electrostatic interactions and hydrogen bonding with amino-functionalized surfaces. The system achieved a DOX loading efficiency of 82.6% at pH 7.4, and adsorption kinetics followed a pseudo-second-order model. *In vitro* release studies demonstrated pronounced pH-dependent behavior, with enhanced drug release under acidic conditions. Upon exposure to an alternating magnetic field (200 Oe, 450 kHz), efficient magnetothermal heating was induced, enabling rapid and externally regulated enhancement of DOX release. Cytotoxicity assays showed negligible intrinsic toxicity of the unloaded carrier under the tested conditions, while the DOX-loaded nanoparticles induced concentration-dependent cytotoxic effects in HepG2 and MCF-7 cells (IC_50_ ≈ 50 µg mL^−1^ for the carrier, corresponding to ∼2.6–2.7 µg mL^−1^ DOX equivalent). AMF-mediated heating resulted in temperature-dependent loss of cell viability, exceeding 90% at 55 °C within 10 min. Overall, the Gd_0.18_Fe_2.82_O_4_@SiO_2_/APTES/DOX system demonstrates alternating magnetic field (AMF)-responsive drug release and pronounced temperature-dependent cytotoxicity, supporting its potential for chemo-magnetic cancer treatment.

## Abstract 2

Externally regulated and stimuli-responsive drug delivery systems remain of significant interest for improving the controllability of cancer treatment strategies.

## Graphical abstract

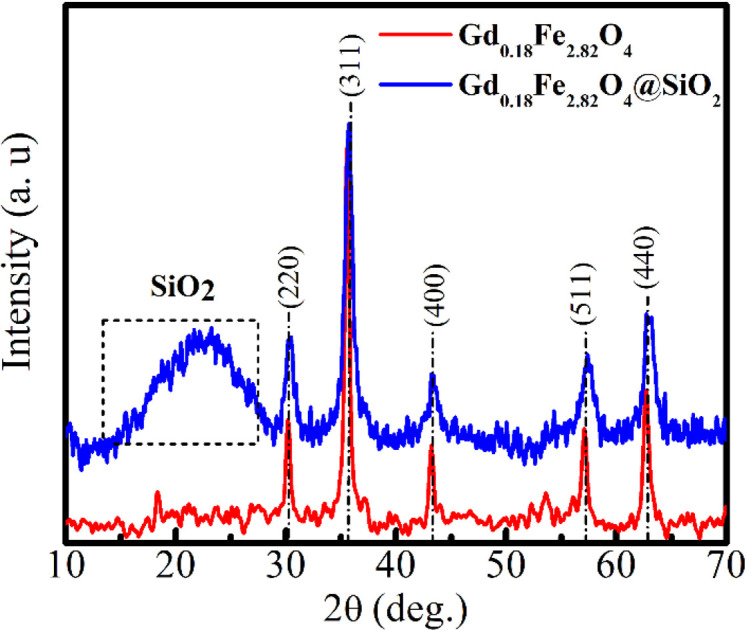

## 1. Introduction

Among the various nanomaterials applied in biomedicine, magnetic nanoparticles (MNPs) have attracted considerable attention owing to their multifunctional capabilities.^1^ In particular, magnetite (Fe_3_O_4_) nanoparticles are widely investigated for biomedical applications such as magnetic resonance imaging (MRI) contrast enhancement, magnetically assisted drug delivery, and the detection of biological species including bacteria, proteins, and viruses.^2^ However, the high surface area-to-volume ratio of Fe_3_O_4_ nanoparticles results in elevated surface energy and a strong tendency toward agglomeration, which compromises colloidal stability and may limit therapeutic performance.^3^ Aggregated nanoparticles are more readily recognized and cleared by macrophages, potentially shortening blood circulation times. Consequently, surface engineering strategies aimed at improving dispersion stability and preserving biodegradability and cytocompatibility have been extensively explored.^4–6^

Among the various surface modification approaches, silica has emerged as an effective shell material for Fe_3_O_4_ nanoparticles due to its favorable cytocompatibility, hydrophilicity, chemical stability, and ease of surface functionalization.^6–8^ In addition, silica shells provide a high density of accessible surface sites, enabling high drug-loading capacity and improved structural stability. Silica coating can also influence the magnetic relaxation behavior of Fe_3_O_4_ nanoparticles, thereby affecting both imaging performance and therapeutic functionality.

Although magnetic metals and alloys often exhibit stronger magnetic responses, their susceptibility to oxidation and potential toxicity significantly restrict their biomedical applicability. In contrast, Fe_3_O_4_ nanoparticles, characterized by superparamagnetism, relatively high saturation magnetization, and favorable biocompatibility profiles reported in the literature, remain among the most suitable candidates for biomedical use.^8,9^ The magnetic properties of Fe_3_O_4_ can be further tailored through lattice doping. In AB_2_O_4_ spinel ferrites, the magnetic response is highly sensitive to the distribution of cations between tetrahedral (A) and octahedral (B) sites; thus, ionic substitution offers an effective route to modulate the inversion degree and engineer tunable magnetic properties.^10,11^ While extensive studies have focused on Fe_3_O_4_ doped with divalent ions such as Mn^2+^, Co^2+^, and Zn^2+^, comparatively fewer investigations have examined Gd-doped Fe_3_O_4_ systems, despite several reports indicating their distinct advantages.

Gd^3+^ ions possess a large magnetic moment (7.9*µ*_B_)^12^ and can substitute for Fe^3+^ ions within the spinel lattice, potentially modifying the overall magnetization of Fe_3_O_4_ nanoparticles. Moreover, gadolinium-based compounds are widely used as MRI contrast agents due to their favorable relaxivity and acceptable toxicity profiles.^13,14^ Recent work by Jiang *et al.*^15^ demonstrated that Gd-doped Fe_3_O_4_ nanoparticles generate higher heat under an alternating magnetic field compared with undoped Fe_3_O_4_, highlighting their potential for magnetic hyperthermia and thermally triggered drug release. In addition, Gd incorporation may contribute to MRI contrast enhancement, enabling imaging-guided monitoring of magnetic nanocarriers in future *in vivo* studies.^15^

Magnetic nanoparticle-based carriers have been extensively explored for the delivery of a broad range of therapeutic agents, including chemotherapeutics (*e.g.*, doxorubicin and paclitaxel), enzymes, anti-inflammatory drugs, folic acid, mitomycin, and nucleic acids.^16^ The therapeutic efficacy of such systems can be further enhanced through the combination of chemotherapy and magnetic hyperthermia, as magnetically induced heating can accelerate drug release and amplify temperature-dependent cytotoxic effects.

In our previous systematic investigation of Fe_3−*x*_Gd_*x*_O_4_ nanoparticles (*x* = 0.00, 0.06, 0.12, 0.18, 0.24, and 0.30),^17^ the composition with *x* = 0.18 exhibited the highest saturation magnetization at room temperature (*M*_s_ = 73.5 emu g^−1^) among the studied series. This enhanced magnetic response suggests a favorable distribution of Gd^3+^ ions within the spinel lattice and indicates strong potential for magnetothermal conversion. Furthermore, in our Fe_3_O_4_@SiO_2_ nanocomposite design, a molar Fe : Si ratio of 0.2 : 1 was previously shown to yield a high specific surface area (*S*_BET_ ≈ 493.6 m^2^ g^−1^),^18^ which is advantageous for drug loading, molecular adsorption, and controlled release.

Based on these considerations, Gd_0.18_Fe_2.82_O_4_@SiO_2_ (Fe : Si = 0.2 : 1) core–shell nanoparticles were selected as a platform combining enhanced magnetic performance with high surface-area-driven drug-loading capability. The silica shell was further functionalized with 3-aminopropyltriethoxysilane (APTES) to introduce abundant surface amine (–NH_2_) groups. These functional groups can interact with the –COOH, –OH, and –C=O moieties of doxorubicin (DOX) through hydrogen bonding, electrostatic interactions, and π–π stacking,^19,20^ thereby enabling efficient drug adsorption and supporting pH-responsive release behavior under acidic conditions relevant to tumor microenvironments.

Beyond physicochemical optimization, biological evaluation is essential for assessing the *in vitro* therapeutic performance of nanocarrier systems. Cytotoxicity studies performed on HepG2 (human hepatocellular carcinoma) and MCF-7 (human breast cancer) cell lines demonstrated that the drug-free Gd_0.18_Fe_2.82_O_4_@SiO_2_/APTES nanocarriers exhibit negligible intrinsic cytotoxicity under the tested conditions. To assess therapeutic functionality, DOX-loaded nanocarriers were further evaluated under an alternating magnetic field. Magnetothermal activation induced substantial temperature-dependent loss of cell viability, particularly at elevated temperatures, indicating a combined effect of hyperthermia and chemotherapeutic drug release.

Overall, this work presents a rationally engineered Gd-doped ferrite core–shell nanoplatform that integrates enhanced magnetic properties, high drug-loading capacity, controlled release behavior, and *in vitro* chemo-hyperthermia activity. By linking magnetic optimization, surface engineering, and biological evaluation, this study contributes to the development of magnetically activatable ferrite-based nanocarriers for externally regulated cancer treatment strategies.

## 2. Experimental

### 2.1. Chemicals

Iron(ii) chloride tetrahydrate (FeCl_2_·4H_2_O, 98%), iron(iii) chloride hexahydrate (FeCl_3_·6H_2_O, 98%), gadolinium(iii) chloride hexahydrate (GdCl_3_·6H_2_O, 99.9%), hydrochloric acid (HCl, 37 wt%), ammonium hydroxide solution (NH_4_OH, 28 wt%), tetraethyl orthosilicate (TEOS, 99.0%), cetyltrimethylammonium bromide (CTAB, 99%), (3-aminopropyl)triethoxysilane (APTES, 99%), ethanol (99.5%), and doxorubicin hydrochloride (DOX, 98%) were purchased from Merck (Germany) and used as received without further purification. Distilled water was used throughout all experiments.

### 2.2. Synthesis of Gd_0.18_Fe_2.82_O_4_@SiO_2_ nanoparticles

#### 2.2.1. Synthesis of Gd_0.18_Fe_2.82_O_4_ core nanoparticles

Gd_0.18_Fe_2.82_O_4_ core nanoparticles were synthesized *via* a conventional co-precipitation method. A homogeneous precursor solution was prepared by dissolving FeCl_2_ (2 mL, 2 M), FeCl_3_ (3.64 mL, 2 M), and GdCl_3_ (3.6 mL, 0.2 M) in 10 mL of 2 M HCl under continuous magnetic stirring. The resulting solution was heated to 60 °C, followed by the dropwise addition of NH_4_OH solution (60 mL, 2 M) to induce alkaline co-precipitation.

Upon completion of the reaction, a black magnetic precipitate was formed and collected using an external magnetic field. The precipitate was thoroughly washed with distilled water until neutral pH was achieved, subsequently rinsed with acetone to remove residual impurities, and dried at 60 °C for 24 h to obtain Gd_0.18_Fe_2.82_O_4_ nanoparticles.

#### 2.2.2. SiO_2_ coating of Gd_0.18_Fe_2.82_O_4_ nanoparticles

Silica coating was performed using a modified sol–gel process in the presence of cetyltrimethylammonium bromide (CTAB) as a structure-directing agent. Briefly, CTAB (2.8 g) was dissolved in a mixed solvent consisting of distilled water (65 mL) and ethanol (20 mL), followed by the addition of NH_4_OH solution (4.4 mL, 25 wt%) to establish an alkaline reaction medium. Gd_0.18_Fe_2.82_O_4_ nanoparticles (0.1 g) were then introduced into the solution and uniformly dispersed by ultrasonication for 20 min.

Subsequently, tetraethyl orthosilicate (TEOS, 1.5 mL) was added dropwise under continuous stirring, and the reaction mixture was maintained at 60 °C for 2 h to allow hydrolysis and condensation of the silica precursor, resulting in the formation of a SiO_2_ shell around the magnetic cores. The resulting Gd_0.18_Fe_2.82_O_4_@SiO_2_ nanoparticles were collected by magnetic separation, thoroughly washed with distilled water, and subjected to Soxhlet extraction with ethanol to remove residual CTAB. The purified product was finally dried at 70 °C for 24 h. The silica-coated sample is hereafter denoted as FG@S.

The selected composition and synthesis parameters were based on prior optimization studies and are discussed in detail in the Introduction.

### 2.3. Surface functionalization of Gd_0.18_Fe_2.82_O_4_@SiO_2_ with APTES

To introduce surface amine (–NH_2_) functional groups, Gd_0.18_Fe_2.82_O_4_@SiO_2_ nanoparticles were functionalized with 3-aminopropyltriethoxysilane (APTES) *via* a silanization reaction. Briefly, 200 mg of Gd_0.18_Fe_2.82_O_4_@SiO_2_ nanoparticles were dispersed in 100 mL of ethanol and ultrasonicated for 15 min to obtain a homogeneous suspension. The dispersion was then magnetically stirred for an additional 15 min, followed by the dropwise addition of APTES (0.5 mL) under continuous stirring.

The reaction mixture was maintained at room temperature for 24 h to allow hydrolysis of the ethoxy groups and subsequent condensation with surface silanol (Si–OH) groups, resulting in covalent grafting of amine functionalities onto the silica shell. The resulting product was collected by filtration, thoroughly washed with distilled water to remove unreacted APTES, and dried at 70 °C for 24 h. The amine-functionalized sample is hereafter denoted as FG@SAP.

### 2.4. Preparation of DOX-loaded nanoparticles

Doxorubicin (DOX) loading was carried out *via* a physical adsorption process. DOX was first dissolved in phosphate-buffered saline (PBS, pH = 7.4) to prepare a stock solution with a concentration of 200 ppm. Subsequently, 20 mg of FG@SAP nanoparticles was dispersed in 10 mL of the DOX solution and shaken at 30 °C for 8 h to facilitate drug adsorption.

The loading process was primarily driven by electrostatic interactions between protonated DOX molecules and surface amine groups, as well as hydrogen bonding and diffusion of DOX into the porous silica shell. After completion of loading, the DOX-loaded nanoparticles were collected by centrifugation, gently washed to remove loosely bound DOX, and freeze-dried to obtain a dry powder. The final DOX-loaded sample is denoted as FG@SAPD.

The selected loading conditions were optimized based on adsorption kinetics analysis, as discussed in Section 3.3.2.

After each synthesis step, nanoparticles were collected by magnetic separation (or centrifugation, if applicable), thoroughly washed with ethanol and deionized water to remove residual reactants, and dried under vacuum at 60 °C for 12 h unless otherwise stated. The dried powders were stored in sealed glass vials at room temperature in a desiccator to prevent moisture adsorption. For short-term biological experiments, nanoparticles were freshly dispersed in sterile phosphate-buffered saline (PBS) or deionized water by ultrasonication prior to use. DOX-loaded nanoparticles were stored in light-protected containers at 4 °C and used within two weeks to minimize potential drug degradation. No apparent changes were observed.

The nanoparticle suspensions were freeze-dried using a laboratory-scale lyophilizer. Samples were first frozen at −80 °C for 12 h to ensure complete solidification. Primary drying was performed at a shelf temperature of −40 °C under a chamber pressure of 0.1 mbar for 24 h to facilitate sublimation of ice. Secondary drying was subsequently conducted at 20 °C under 0.01 mbar for 8 h to remove residual bound moisture. The total freeze-drying cycle duration was approximately 44 h. The obtained dry powders were immediately sealed in airtight glass vials and stored in a desiccator until further use. A representative photograph of the final freeze-dried product is provided in Fig. S1.

### 2.5. Physicochemical characterization

The crystal structure of the synthesized materials was analyzed by X-ray diffraction (XRD) at room temperature using Cu Kα radiation (*λ* = 1.5406 Å) on a Bruker D8 Advance diffractometer. Chemical bonding and surface functional groups were examined by Fourier-transform infrared (FT-IR) spectroscopy using a PerkinElmer Spectrum Two spectrometer. UV-Vis absorption spectra were recorded on a Jasco V-670 spectrophotometer to quantify doxorubicin (DOX) loading and to monitor drug release behavior.

The surface morphology and particle size were examined using a field-emission scanning electron microscope (FE-SEM, HITACHI S-4800) equipped with an energy-dispersive X-ray spectroscopy (EDX) detector for elemental analysis. The internal structure, core–shell architecture, and detailed morphology of the nanoparticles were further characterized by transmission electron microscopy (TEM, JEOL JEM-2100Plus) operated at an accelerating voltage of 200 kV.

The hydrodynamic diameter, polydispersity index (PDI), and colloidal stability of the samples were determined by dynamic light scattering (DLS), while surface charge was evaluated by zeta potential measurements using a Malvern Zetasizer. The specific surface area, pore volume, and pore size distribution were measured by nitrogen adsorption–desorption isotherms using a TriStar II Plus analyzer, and the Brunauer–Emmett–Teller (BET) method was applied for surface area calculations.

Magnetic properties were evaluated using a vibrating sample magnetometer (VSM) with an applied magnetic field ranging from −20 to 20 kOe. Induction heating performance under an alternating magnetic field was investigated using a high-frequency induction heating system (RDO-HFI, 5 kW).

These characterization techniques were employed to explore correlations between structural, surface, magnetic, and physicochemical properties and the drug loading capacity, release behavior, and AMF-responsive *in vitro* performance.

### 2.6. Adsorption and AMF-responsive desorption of doxorubicin

To investigate the adsorption behavior and AMF-responsive desorption of Doxorubicin (DOX), DOX-loaded nanoparticles (FG@SAPD) were subjected to adsorption–desorption experiments under an alternating magnetic field (AMF). Briefly, 30 mg of FG@SAPD nanoparticles were dispersed in 10 mL of phosphate-buffered saline (PBS, pH 7.4) and ultrasonicated to obtain a homogeneous suspension.

For AMF-responsive desorption experiments, the suspension was exposed to an alternating magnetic field (250 Oe, 450 kHz). Temperature was continuously monitored using a fiber-optic probe inserted into the nanoparticle suspension, reflecting bulk temperature changes of the system. AMF exposure parameters were adjusted accordingly to reach and maintain the desired equilibrium temperatures (38, 42, 47, and 52 °C). At predetermined time intervals, aliquots were withdrawn, magnetically separated, and the supernatant was analyzed by UV-Vis spectroscopy to quantify released DOX. Control experiments were conducted under identical conditions in the absence of AMF to evaluate passive diffusion-driven release. The desorption behavior is interpreted in relation to the experimentally measured bulk temperature variation under AMF exposure. Therefore, the observed release enhancement is attributed to thermally mediated effects under AMF exposure. No direct quantification of nanoscale interfacial temperature was performed in this study.

Detailed experimental protocols, calibration procedures, and data analysis methods are provided in the SI.

### 2.7. Cytotoxicity assay and cellular response evaluation

Human hepatocellular carcinoma (HepG2) and human breast adenocarcinoma (MCF-7) cell lines were employed to evaluate the *in vitro* cytotoxicity and cellular responses induced by the synthesized nanomaterials. Both cell lines were originally obtained from the American Type Culture Collection (ATCC) and were maintained by the Experimental Oncology Research Group, Department of Cell Biology, Faculty of Biology, University of Science, Vietnam National University, Hanoi.

#### 2.7.1. Cell culture

Cryopreserved HepG2 and MCF-7 cells were rapidly thawed in a 37 °C water bath and centrifuged at 1300 rpm for 5 min to remove the cryoprotectant. The resulting cell pellets were resuspended in the appropriate complete culture medium, consisting of Dulbecco's Modified Eagle Medium (DMEM) for HepG2 cells or RPMI-1640 medium for MCF-7 cells, each supplemented with 10% fetal bovine serum (FBS) and 1% penicillin–streptomycin.

Cells were seeded into culture flasks and incubated at 37 °C in a humidified atmosphere containing 5% CO_2_. The culture medium was replaced every two days, and cells were subcultured at approximately 60–70% confluence until sufficient cell numbers were obtained for subsequent cytotoxicity experiments.

#### 2.7.2. *In vitro* cytotoxicity assay

The cytotoxic effects of the prepared nanomaterials on HepG2 and MCF-7 cells were evaluated using a colorimetric cell viability assay based on mitochondrial metabolic activity (CellTiter 96® Non-Radioactive Cell Proliferation Assay, Promega). Due to the solubility characteristics of the tested samples, nanoparticle dispersions were prepared in phosphate-buffered saline (PBS) containing 0.15% acetic acid. The concentration ranges investigated in the cytotoxicity assays are summarized in Table S2 (SI).

Cells were seeded into 96-well plates at a density of 5 × 10^3^ cells per well in 180 µL of complete culture medium and allowed to adhere for 24 h at 37 °C under 5% CO_2_. Subsequently, 20 µL of nanoparticle dispersions at various concentrations were added to each well, and the cells were incubated for an additional 48 h.

After treatment, 20 µL of the assay reagent was added to each well, followed by incubation for 4 h in the dark to allow the formation of the colored formazan product. The reaction was terminated by adding 100 µL of the stop/solubilization solution, and the plates were incubated for a further 1 h to ensure complete color development. Absorbance was measured at 490 nm using a microplate reader.

Cell viability was calculated as a percentage relative to untreated control cells according to the following equation:1
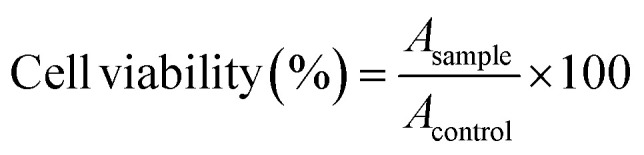
where *A*_sample_ and *A*_control_ represent the average absorbance values of treated and untreated cells, respectively. The half-maximal inhibitory concentration (IC_50_) was defined as the concentration of nanomaterial required to reduce cell viability to 50% of the control value.

### 2.8. *In vitro* magnetothermal treatment of MCF-7 cells

The *in vitro* magnetothermal therapeutic performance of DOX-loaded FG@SAPD nanoparticles was evaluated using human breast cancer MCF-7 cells. Cell culture conditions and maintenance procedures were identical to those described in Section 2.7. For magnetothermal treatment, a cell suspension (750 µL) containing 2 × 10^6^ MCF-7 cells was mixed with 250 µL of FG@SAPD nanoparticle dispersion at a concentration corresponding to half of the IC_50_ value (25 µg mL^−1^). The resulting cell–nanoparticle mixtures were exposed to an alternating magnetic field (AMF) for 5 or 10 min. Bulk temperatures of approximately 45 °C and 55 °C were reached after 5 and 10 min of AMF exposure, respectively. Temperature was continuously monitored at the culture medium level using a fiber-optic probe, reflecting bulk heating conditions of the system. Appropriate control groups were included to differentiate intrinsic nanocarrier cytotoxicity from AMF-associated heating effects. These controls comprised cells treated with phosphate-buffered saline (PBS, 1×) and cells incubated with FG@SAPD nanoparticles in the absence of AMF exposure. Following AMF treatment, all samples were incubated under standard culture conditions, and cell viability was assessed after 24 h using the colorimetric assay described in Section 2.7. A detailed overview of the experimental design and control groups is provided in Table S3 (SI).

Intracellular or particle–cell interface temperature was not directly quantified in this study. Therefore, the observed biological responses are interpreted in relation to bulk temperature elevation under AMF exposure rather than directly measured intracellular hyperthermia.

## 3. Results and discussion

### 3.1. Structural, optical, magnetic, and magnetothermal characterization

**Fig. 1:**
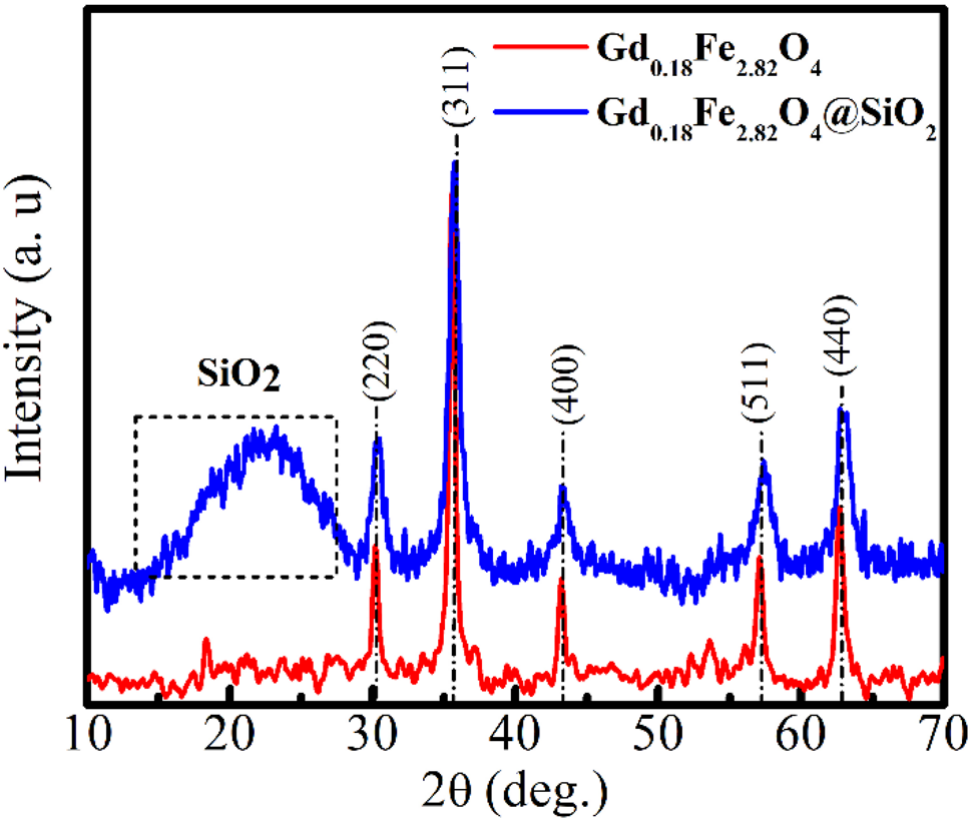
XRD patterns of Gd_0.18_Fe_2.82_O_4_ and Gd_0.18_Fe_2.82_O_4_@SiO_2_ nanoparticles.

Fig. 1 presents the X-ray diffraction (XRD) patterns of the Gd_0.18_Fe_2.82_O_4_ core nanoparticles and the corresponding Gd_0.18_Fe_2.82_O_4_@SiO_2_ core–shell nanocomposite. For both samples, the diffraction peaks located at 2*θ* ≈ 30.3°, 35.7°, 43.5°, 57.1°, and 62.9° can be indexed to the (220), (311), (400), (511), and (440) crystallographic planes of the cubic spinel structure, in good agreement with standard magnetite (Fe_3_O_4_) (PDF No. 00-001-1111). No additional crystalline reflections associated with impurity phases or gadolinium-containing oxides are detected, indicating that Gd^3+^ incorporation occurs within the spinel lattice without altering the single-phase crystal structure.

For the Gd_0.18_Fe_2.82_O_4_@SiO_2_ sample, a broad diffuse scattering feature in the 2*θ* range of approximately 10–30° is observed, which is characteristic of amorphous silica (JCPDS No. 29-0085). The coexistence of sharp spinel diffraction peaks from the ferrite core and the amorphous halo from the silica shell confirms the successful formation of the core–shell Gd-doped ferrite@SiO_2_ nanostructure. Similar diffraction characteristics have been widely reported for silica-coated spinel ferrite systems, where the amorphous SiO_2_ shell does not disrupt the crystalline core structure.^21–25^

**Fig. 2:**
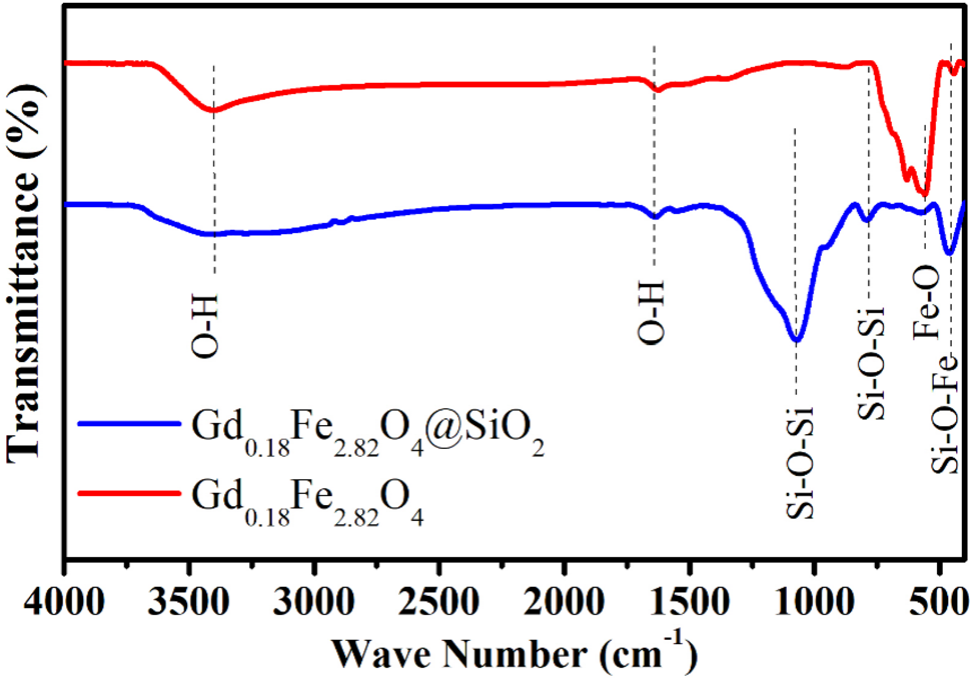
FTIR spectra of Gd_0.18_Fe_2.82_O_4_ and Gd_0.18_Fe_2.82_O_4_@SiO_2_ confirming the presence of Si–O–Si, Fe–O, and Si–O–Fe bonds indicative of a core–shell architecture.

To further elucidate the chemical structure and verify the formation of the core–shell architecture, the FTIR spectra of Gd_0.18_Fe_2.82_O_4_ and Gd_0.18_Fe_2.82_O_4_@SiO_2_ (FG@S) nanoparticles were analyzed, as shown in Fig. 2. Both samples exhibit a broad absorption band in the range of 3300–3700 cm^−1^, which is attributed to O–H stretching vibrations of surface-adsorbed hydroxyl groups, while the band near 1630 cm^−1^ corresponds to the H–O–H bending mode of physically adsorbed or coordinated water molecules.^26^

In the core–shell sample, characteristic vibrational features associated with the silica shell are clearly observed. The strong band centered at approximately 1070 cm^−1^ and the band at around 784 cm^−1^ are assigned to the asymmetric and symmetric stretching vibrations of Si–O–Si bonds, respectively, whereas the band near 570 cm^−1^ is attributed to Si–O–Si bending vibrations, confirming the formation of an amorphous silica network on the nanoparticle surface.^27^ Concurrently, the Fe–O stretching vibration of the ferrite core, typically observed at approximately 628 cm^−1^, exhibits a marked reduction in intensity after silica coating, which can be ascribed to partial shielding of the magnetic core by the SiO_2_ shell.^28^

Both samples also display an absorption band near 462 cm^−1^. For the bare Gd_0.18_Fe_2.82_O_4_ nanoparticles, this band mainly arises from Fe–O bending vibrations associated with the octahedral sites of the spinel lattice. In contrast, its enhanced intensity in the SiO_2_-coated sample likely reflects an additional contribution from interfacial Si–O–Fe linkages, which are commonly reported to appear in the 450–500 cm^−1^ region for ferrite–silica hybrid systems. This spectral feature therefore supports the formation of chemical bonding at the ferrite–silica interface.

Furthermore, a slight shift of the H–O–H bending band from 1632 to 1619 cm^−1^ is observed after silica deposition, which can be associated with hydrogen-bonding interactions between surface silanol (Si–OH) groups and hydroxylated sites on the Gd_0.18_Fe_2.82_O_4_ nanoparticles.^29^ Overall, the FTIR results are in good agreement with previously reported ferrite@SiO_2_ nanocomposites and corroborate the successful synthesis of the intended core–shell nanostructure.^30,31^

**Fig. 3:**
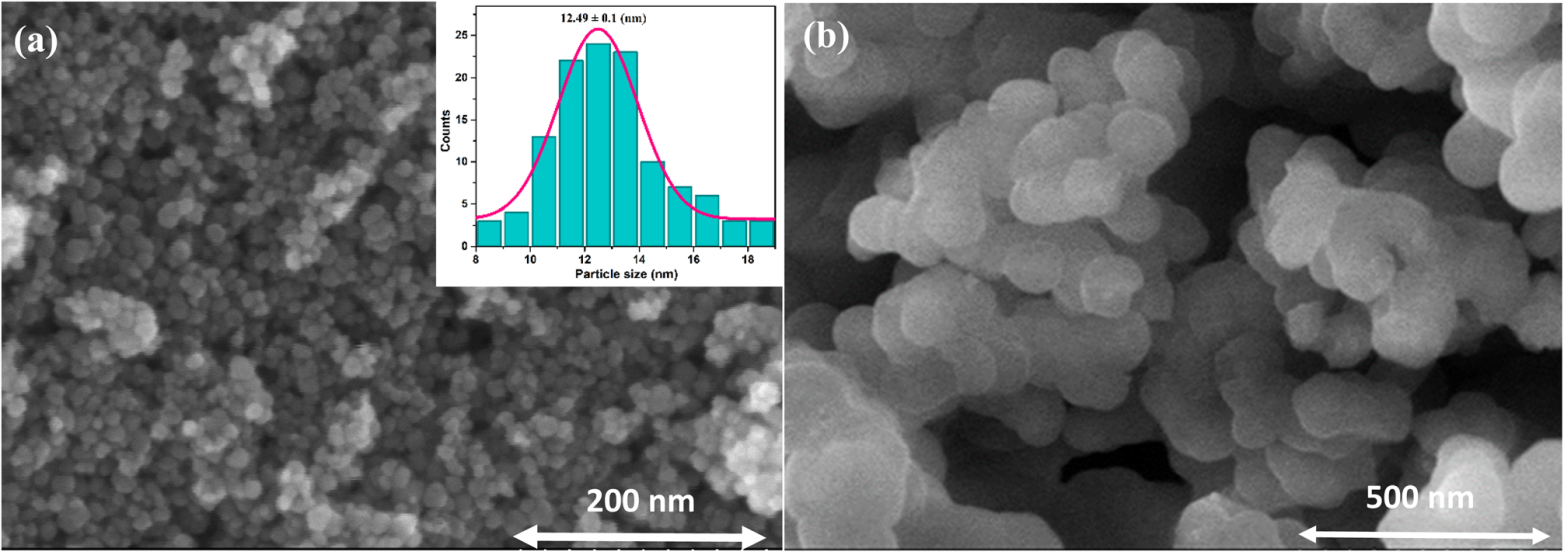
FESEM images of (a) Gd_0.18_Fe_2.82_O_4_ and (b) Gd_0.18_Fe_2.82_O_4_@SiO_2_ nanoparticles. The inset in (a) shows the particle size distribution of Gd_0.18_Fe_2.82_O_4_.

Field-emission scanning electron microscopy (FESEM) was employed to examine the morphology and size evolution of the nanoparticles before and after silica coating, as shown in Fig. 3. The pristine Gd_0.18_Fe_2.82_O_4_ nanoparticles (Fig. 3a) exhibit a predominantly spherical morphology with clearly discernible particle boundaries and a relatively narrow size distribution. A certain degree of agglomeration is observed, which is commonly attributed to magnetic dipole–dipole interactions among ferrite nanoparticles. Owing to the well-resolved particle contours, statistical analysis of the particle size distribution was performed (inset of Fig. 3a). The particle diameters range from approximately 8 to 16 nm, with an average size of about 12.5 nm. This nanoscale dimension is favorable for use as a magnetic core in core–shell nanostructures and is particularly relevant for biomedical and magnetothermal applications.

Following silica coating, the Gd_0.18_Fe_2.82_O_4_@SiO_2_ nanoparticles (Fig. 3b) largely preserve a spherical morphology; however, the particle boundaries become noticeably less distinct compared to those of the uncoated ferrite cores. This feature is characteristic of silica shells formed *via* Stöber-type sol–gel processes,^32^ in which the hydrolysis and condensation of tetraethyl orthosilicate (TEOS) generate an amorphous and continuous SiO_2_ layer with low electron contrast. As a result, individual core–shell particles are not sharply resolved in FESEM images, particularly when the silica shell thickness is sufficient to obscure the underlying magnetic core.

Due to the indistinct particle boundaries and the amorphous nature of the silica shell, a reliable particle size distribution could not be extracted from the FESEM images of the Gd_0.18_Fe_2.82_O_4_@SiO_2_ sample. Nevertheless, the apparent increase in particle dimensions relative to the pristine ferrite cores, together with the loss of visible core features, qualitatively indicates effective silica encapsulation. Similar FESEM observations have been widely reported for ferrite@SiO_2_ core–shell systems synthesized *via* seeded Stöber routes, for which more accurate size information is typically obtained from TEM analysis or hydrodynamic measurements.

Overall, the FESEM results demonstrate the morphological evolution from discrete ferrite nanoparticles to silica-encapsulated core–shell nanostructures, providing a suitable foundation for subsequent surface functionalization and biomedical application.^33^

**Fig. 4:**
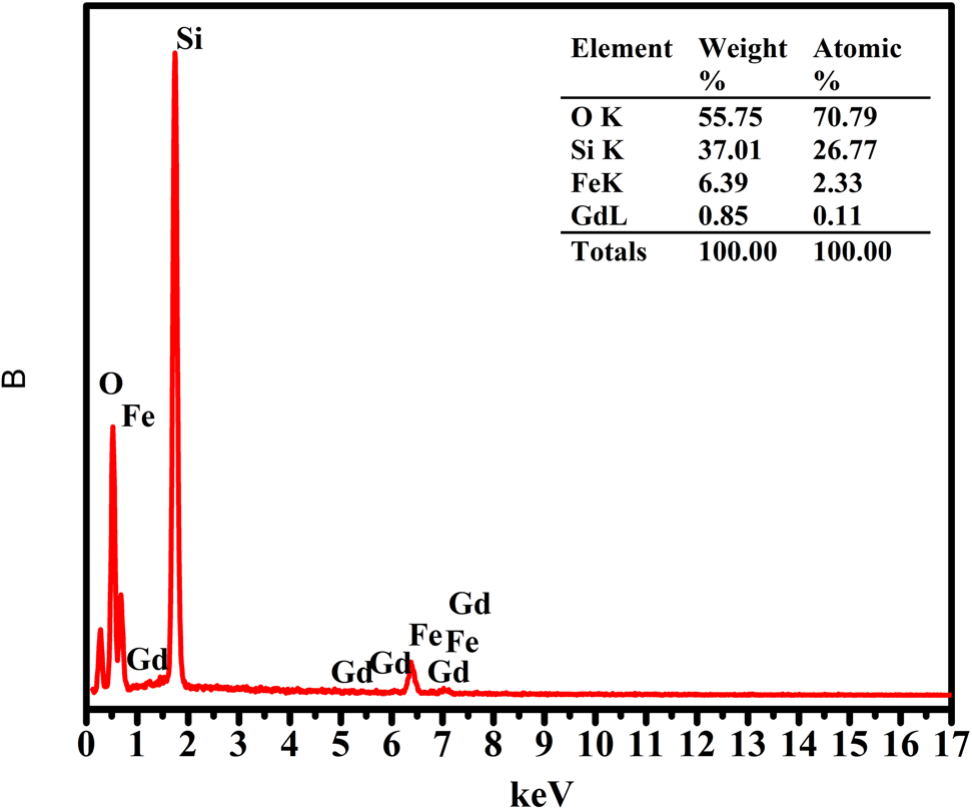
EDX spectra of Gd_0.18_Fe_2.82_O_4_@SiO_2_ nanoparticles.

To further verify the presence of the SiO_2_ shell and examine the elemental composition of the nanoparticles, energy-dispersive X-ray (EDX) analysis was carried out, as shown in Fig. 4. For the pristine ferrite sample, only Fe and Gd signals are detected, in agreement with the expected elemental composition of the Gd-doped ferrite core.

In contrast, the FG@S sample exhibits a pronounced Si signal, accompanied by a substantial increase in the relative Si content compared to those of Fe and Gd. The estimated Si mass fraction (∼37%) is markedly higher than those of Fe (6.39%) and Gd (0.85%), indicating the dominance of silica in the probed volume. While EDX provides semi-quantitative information, the strong Si signal, together with the simultaneous detection of Fe, Gd, O, and Si, clearly evidences effective silica coating and the coexistence of core and shell constituents within the same nanostructure.

These elemental features are consistent with the formation of a ferrite@SiO_2_ core–shell architecture and corroborate the structural and spectroscopic results discussed above.

**Fig. 5:**
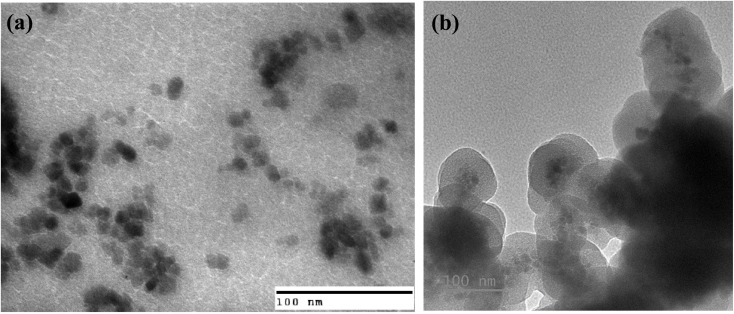
TEM images of (a) bare Gd_0.18_Fe_2.82_O_4_ nanoparticles and (b) Gd_0.18_Fe_2.82_O_4_@SiO_2_ nanocomposite, showing small ferrite cores (8–16 nm) embedded within larger SiO_2_ spheres (60–140 nm) with an average shell thickness of ∼26 nm.

To directly visualize and verify the core–shell architecture, transmission electron microscopy (TEM) was performed, as shown in Fig. 5. The pristine Gd_0.18_Fe_2.82_O_4_ nanoparticles appear as well-dispersed, high-contrast dark particles with sizes in the range of 8–16 nm, in good agreement with the FESEM observations.

In the silica-coated sample, the ferrite cores are clearly encapsulated within larger, low-contrast amorphous SiO_2_ matrices. The distinct contrast difference between the dense magnetic core and the surrounding silica shell allows direct identification of the core–shell configuration. The overall particle sizes range from approximately 60 to 140 nm, while the SiO_2_ shell thickness is estimated to be ∼26 nm on average. Minor variations in shell thickness are observed, which are typical for Stöber-derived silica coatings.

These TEM observations provide direct structural evidence for the successful formation of ferrite@SiO_2_ core–shell nanoparticles and are fully consistent with the morphological features inferred from FESEM and the elemental composition confirmed by EDX analysis.

**Fig. 6:**
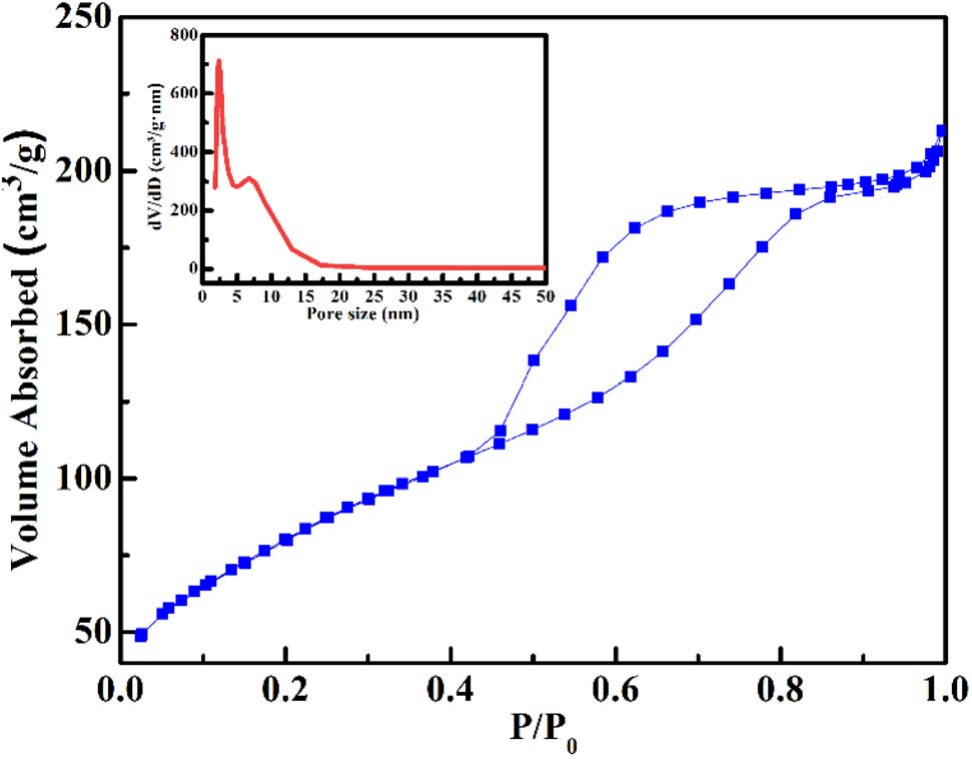
N_2_ adsorption–desorption isotherms (type IV with H_3_ hysteresis) and corresponding pore-size distribution (inset) of the Gd_0.18_Fe_2.82_O_4_@SiO_2_ nanocomposite.

BET surface area and N_2_ adsorption–desorption measurements were carried out to evaluate the surface textural properties of the FG@S nanocomposite, which are critical for its drug-loading performance. As shown in Fig. 6, the adsorption–desorption isotherm corresponds to type IV according to the IUPAC classification, with a pronounced H_3_ hysteresis loop, indicative of mesoporous structures arising from the aggregation of amorphous silica domains. The appearance of the H_3_ loop at relative pressures *P*/*P*_0_ > 0.4 is associated with capillary condensation within slit-shaped interparticle mesopores, a feature commonly observed in silica-based mesoporous systems.^34^

The FG@S nanocomposite exhibits a high specific surface area of 300 m^2^ g^−1^, a total pore volume of 0.354 cm^3^ g^−1^, and a broad pore-size distribution spanning 1.5–15 nm, with a dominant mesopore diameter centered at approximately 4.7 nm. Such mesoporous channels are sufficiently large to facilitate the diffusion and adsorption of doxorubicin molecules (≈1 nm), thereby favoring efficient drug loading.^35,36^

**Fig. 7:**
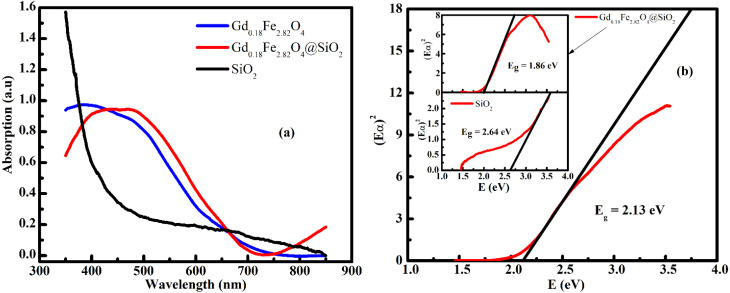
(a) UV-Vis absorption spectra of Gd_0.18_Fe_2.82_O_4_, Gd_0.18_Fe_2.82_O_4_@SiO_2_, and SiO_2_. (b) Tauc plot assuming direct allowed transitions for Gd_0.18_Fe_2.82_O_4_; the inset shows the corresponding Tauc plots for the Gd_0.18_Fe_2.82_O_4_@SiO_2_ nanocomposite (direct) and SiO_2_ (indirect).

The UV-Vis absorption spectra of Gd_0.18_Fe_2.82_O_4_, FG@S, and SiO_2_ are presented in Fig. 7. The bare ferrite nanoparticles exhibit a broad absorption onset around ∼400 nm, which is attributed to O(2p) → Fe(3d) charge-transfer transitions characteristic of spinel ferrites.^37^ In contrast, amorphous SiO_2_ shows negligible absorption in the visible region, consistent with its wide-band-gap insulating nature.^38^ After silica encapsulation, the FG@S nanocomposite does not display a distinct absorption peak but instead exhibits a broadened absorption edge with a visible-light absorption tail extending into the 400–500 nm region. This behavior reflects a modified optical response induced by the core–shell architecture, arising from interfacial disorder, microstrain, and the formation of Si–O–Fe bonds at the ferrite–silica interface, rather than from intrinsic absorption of the silica shell.^38^

To further quantify the optical response, the band gaps were estimated using Tauc plots derived from the UV-Vis spectra.^39^ A direct allowed transition model was applied for Gd_0.18_Fe_2.82_O_4_ and FG@S, in accordance with previous reports on nanoscale spinel ferrites, whereas an indirect transition model was employed for amorphous SiO_2_. The extracted optical band gap of bare Gd_0.18_Fe_2.82_O_4_ is approximately 2.13 eV, while SiO_2_ exhibits a significantly larger value of ∼2.64 eV, reflecting its electronically insulating character. Notably, the FG@S nanocomposite shows a reduced effective optical band gap of ∼1.86 eV, corresponding to a weak red shift relative to the pristine ferrite core.

This apparent band-gap narrowing should be interpreted as an effective optical response rather than a true modification of the intrinsic electronic band structure of the ferrite phase. The observed red shift can be reasonably ascribed to interface-induced defect states, lattice distortion, and electronic perturbations at the ferrite–silica boundary, as well as light-scattering effects associated with the core–shell morphology. Similar trends have been widely reported for ferrite@SiO_2_ nanocomposites and are commonly attributed to interfacial and structural effects rather than genuine band-gap renormalization. Overall, the UV-Vis and band-gap analyses collectively confirm the successful formation of the core–shell structure and highlight the role of the ferrite–silica interface in modulating the optical response of the nanocomposite.

**Fig. 8:**
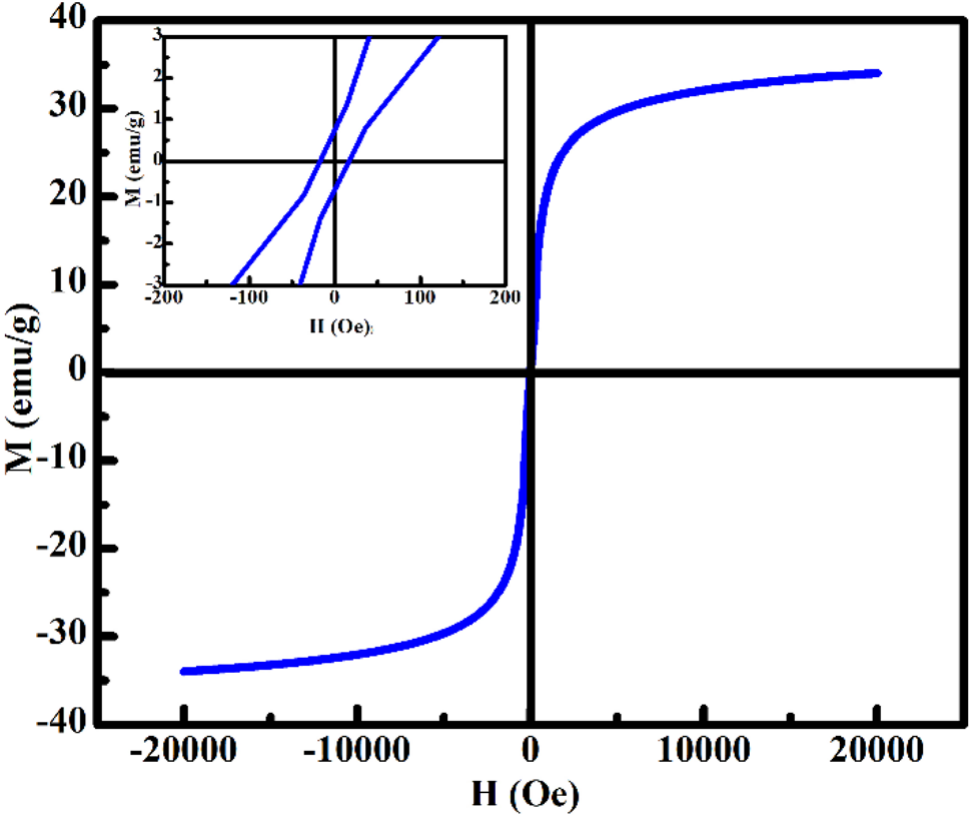
Room-temperature magnetization as a function of applied magnetic field for the Gd_0.18_Fe_2.82_O_4_@SiO_2_ nanocomposite. The inset shows an enlarged view of the low-field region, highlighting the coercivity and remanent magnetization.

The room-temperature magnetization curve (M–H) of FG@S is presented in Fig. 8. The nanocomposite exhibits a saturation magnetization (*M*_s_) of approximately 34 emu g^−1^, which is substantially lower than that of the bare Gd_0.18_Fe_2.82_O_4_ ferrite core (73.5 emu g^−1^) reported previously.^17^ This reduction is primarily attributed to the magnetic dilution effect introduced by the non-magnetic SiO_2_ shell, as well as partial surface spin disorder at the ferrite–silica interface. Despite this decrease, the *M*_s_ value of FG@S remains notably higher than those typically reported for conventional Fe_3_O_4_@SiO_2_ systems,^31,40–42^ underscoring the beneficial contribution of Gd^3+^ substitution to the magnetic response of the ferrite core.

A slight increase in coercivity is observed, from 9 Oe for the bare ferrite^17^ to approximately 16 Oe for the FG@S nanocomposite. This enhancement can be reasonably ascribed to increased interfacial anisotropy and spin pinning effects arising from the ferrite–silica boundary. Importantly, the very low remanence-to-saturation magnetization ratio (*M*_r_/*M*_s_ ≈ 0.018) confirms the superparamagnetic-like behavior of the nanocomposite at room temperature.^11^ Such magnetic characteristics are highly desirable for biomedical applications, as they ensure rapid magnetic response under an external field while minimizing residual magnetization and particle aggregation after field removal.

**Fig. 9:**
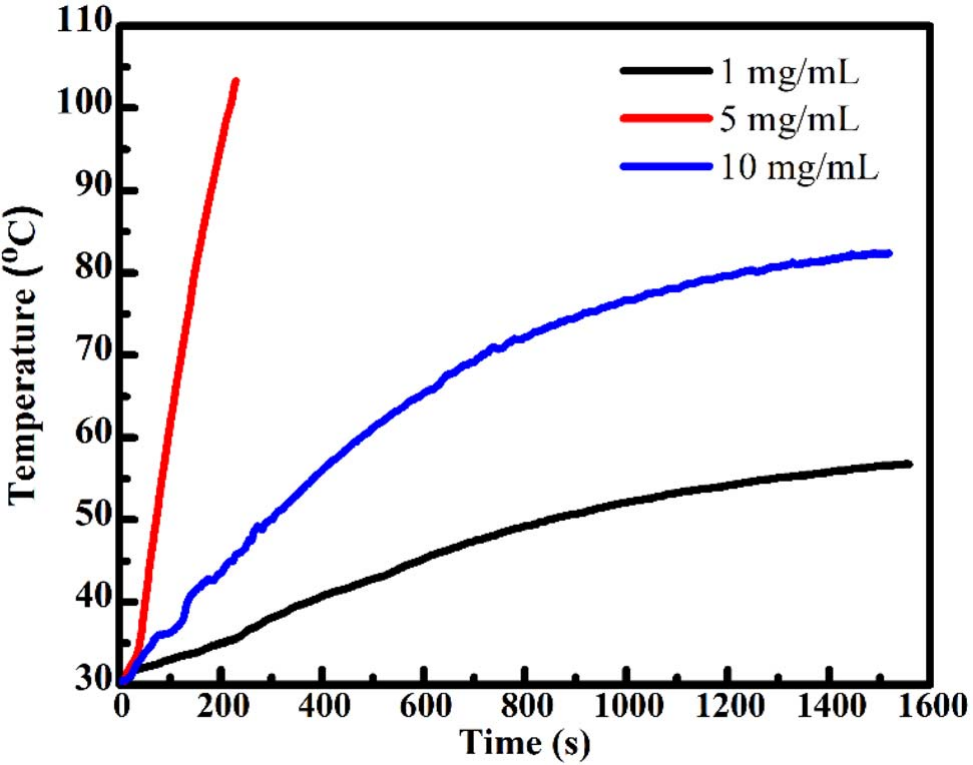
Magnetic hyperthermia heating profiles of Gd_0.18_Fe_2.82_O_4_@SiO_2_ nanocomposites at various concentrations under an alternating magnetic field (200 Oe, 450 kHz), used to evaluate the specific absorption rate (SAR).

To further evaluate the applicability of FG@S as a multifunctional nanoplatform for magnetically guided drug delivery combined with magnetic hyperthermia, its heat-generation capability under an alternating magnetic field (AMF) was investigated. Fig. 9 presents the temperature–time profiles recorded at an AMF amplitude of 200 Oe and a frequency of 450 kHz for aqueous dispersions with particle concentrations of 1, 5, and 10 mg mL^−1^.

The specific absorption rate (SAR) was calculated from the initial heating slope (d*T*/d*t*) using eqn (2) and (3):^11^2
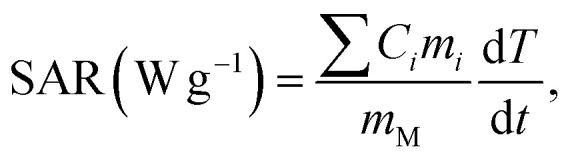
where *C*_*i*_ and *m*_*i*_ are the specific heat capacities and masses of the core–shell components of the FG@S system. For the Gd_0.18_Fe_2.82_O_4_-doped sample, the specific heat capacities were approximated as those of Fe_3_O_4_ nanoparticles (*C*_Fe3O4_ = 0.65 J g^−1^ K^−1^), SiO_2_ (*C*_SiO_2__ = 0.680 J g^−1^ K^−1^), and water (*C*_H_2_O_ = 4.185 J g^−1^ K^−1^). *m*_M_ is the mass of the FG@S nanoparticles, and *m*_H_2_O_ is the mass of the aqueous solution, calculated according to the known core–shell ratio. d*T*/d*t* is the initial rate of temperature increase, determined from the time-dependent temperature profile:

3 *T*(*t*) = *T*_initial_ + Δ*T*_max_[1 − exp(−*t*/*τ*)],

where Δ*T*_max_ is the highest temperature difference from the time the magnetic field is applied and the temperature at the time of measurement, *τ* is the time constant. The value d*T*/d*t* = Δ*T*_max_/*τ* is determined through data matching *via*eqn (3).

The obtained SAR values are 153, 92, and 79 W g^−1^ for concentrations of 1, 5, and 10 mg mL^−1^, respectively. The observed decrease in SAR with increasing concentration is commonly attributed to enhanced interparticle magnetic interactions and agglomeration effects, which suppress Néel and Brownian relaxation mechanisms responsible for efficient heat dissipation.

Notably, the SAR value of 153 W g^−1^ at 1 mg mL^−1^ compares favorably with, and in several cases exceeds, those reported for Fe_3_O_4_@SiO_2_-based systems under similar field conditions.^19–22,24^ The enhanced heating efficiency can be ascribed to the combined effects of Gd^3+^ substitution in the ferrite lattice, which modifies magnetic anisotropy and relaxation dynamics, and the SiO_2_ shell, which improves colloidal stability and heat transfer to the surrounding medium.

Taken together, the optimized core–shell FG@S nanocomposite exhibits a suitable size (∼100.9 nm), high specific surface area (300 m^2^ g^−1^), superparamagnetic behavior with moderate saturation magnetization (∼34 emu g^−1^), and efficient AMF-induced heating, thereby fulfilling the essential material requirements for magnetically responsive drug delivery and hyperthermia-assisted cancer therapy. Building upon the structural and magnetic characterization of the FG@S platform, the present section investigates the functional performance of the APTES-modified, DOX-loaded nanocomposite (FG@SAPD), with particular emphasis on drug-loading capability, magnetically triggered release behavior, and *in vitro* anticancer efficacy.

### 3.2. Morphology, structure, and surface properties of DOX-loaded FG@SAPD nanoparticles

#### 3.2.1. Morphology and particle size of DOX-loaded FG@SAP nanoparticles

**Fig. 10:**
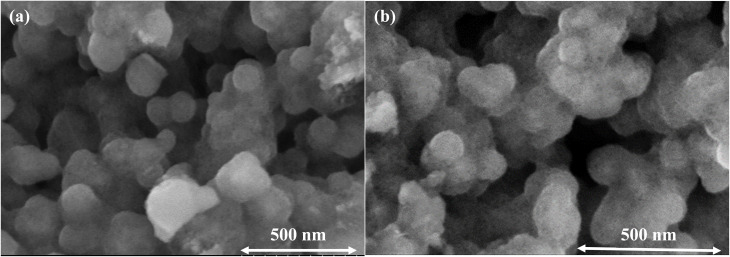
SEM images of APTES-modified Gd_0.18_Fe_2.82_O_4_@SiO_2_ nanoparticles after doxorubicin loading (FG@SAPD) at different drug-loading efficiencies: (a) 85% and (b) 96.7%.

Fig. 10 shows SEM images of APTES-modified FG@SAP nanoparticles after doxorubicin loading (FG@SAPD) at two representative drug-loading efficiencies: (a) 85% and (b) 96.7%. The nanoparticles largely retain a quasi-spherical morphology with well-defined boundaries, although partial agglomeration is observed. Particle sizes range from ∼90 to 160 nm. Compared with pristine FG@SAP, FG@SAPD exhibits enhanced surface contrast and a thin organic layer surrounding the particles, consistent with DOX adsorption on the surface and within the mesoporous silica shell. The darker contrast in SEM images aligns with the organic nature of DOX, as reported for other drug-loaded inorganic nanocarriers.

To assess their hydrodynamic behavior under physiological conditions, FG@SAPD suspensions were prepared in PBS (pH 7.4) by dispersing 5 mg nanoparticles in 40 mL buffer, followed by 10 min ultrasonication and 20 min mechanical stirring. Hydrodynamic particle sizes were measured by dynamic light scattering (DLS), as discussed in the next section.

#### 3.2.2. Dynamic light scattering (DLS) and zeta potential analysis

**Fig. 11:**
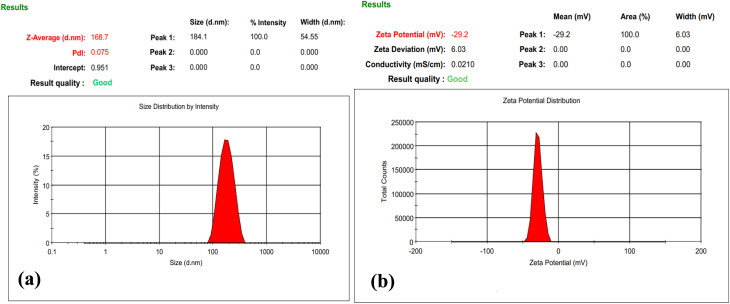
(a) Hydrodynamic particle size distribution and (b) zeta potential of DOX-loaded FG@SAPD nanoparticles measured in PBS buffer (pH 7.4).

Fig. 11 shows the hydrodynamic size distribution and zeta potential of DOX-loaded FG@SAPD nanoparticles dispersed in PBS (pH 7.4). As shown in Fig. 11a, FG@SAPD exhibits a monomodal distribution with an average hydrodynamic diameter of 168.7 nm, and most particles fall within ∼80–400 nm. The polydispersity index (PDI) is 0.075, indicating a narrow size distribution and excellent colloidal dispersion.^43^

Compared with SEM measurements (Fig. 10, 90–140 nm), the slightly larger hydrodynamic diameter is expected, reflecting the solvation layer, surface-bound DOX, and APTES-functionalized silica shell in PBS. The close agreement between SEM and DLS confirms good dispersion stability under physiological conditions.

Fig. 11b shows the zeta potential of FG@SAPD nanoparticles at −29.2 mV. This high absolute value, near the ±30 mV threshold, indicates strong electrostatic repulsion and colloidal stability in suspension,^44^ which is critical for drug delivery and *in vitro* biomedical studies.

#### 3.2.3. FTIR analysis of DOX-loaded FG@SAPD nanoparticles

**Fig. 12:**
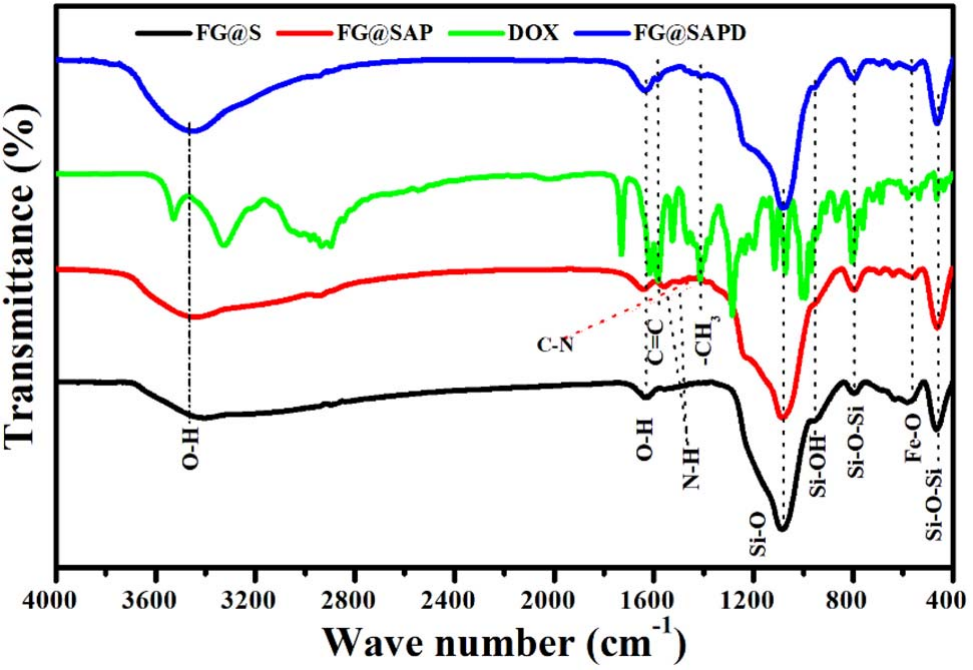
FTIR spectra of FG@S, FG@SAP, FG@SAPD, and pristine DOX, confirming APTES functionalization and successful DOX adsorption.

Fig. 12 presents the FTIR spectra of FG@S, APTES-modified FG@S (FG@SAP), DOX-loaded FG@SAP (FG@SAPD), and pristine DOX. In FG@SAP, the band at ∼1386 cm^−1^ corresponds to C–N stretching, while bands at 1496 and 1559 cm^−1^ arise from N–H bending of amino groups introduced by APTES functionalization.^45^ The Si–OH vibration at ∼960 cm^−1^, prominent in FG@S, decreases markedly after APTES modification, indicating successful amino grafting onto the silica surface.^46^

After DOX loading, FG@SAPD retains all characteristic FG@SAP bands, with additional features from DOX. A weak band at ∼1583 cm^−1^ is assigned to aromatic C=C stretching, confirming DOX presence on the nanoparticle surface.^47^ The broad region between 1600–1700 cm^−1^ shows increased intensity due to overlapping O–H bending and aromatic C=C stretching from DOX,^46^ suggesting strong intermolecular interactions rather than new covalent bond formation. The band at ∼1412 cm^−1^, attributed to methyl bending in DOX,^45^ further supports successful drug adsorption.

Overall, these spectral features indicate that DOX is effectively adsorbed onto APTES-functionalized FG@SAP nanoparticles *via* electrostatic interactions and hydrogen bonding, favorable for pH-responsive and magnetically triggered drug release under physiological conditions.

### 3.3. Adsorption and magnetically triggered desorption of DOX

In this study, DOX adsorption was performed under static conditions without a magnetic field to evaluate the intrinsic drug-loading capacity of the nanocomposite. Drug-release behavior was then investigated both in the absence and presence of an alternating magnetic field to assess magnetically triggered release.

#### 3.3.1. DOX adsorption behavior under static conditions

**Fig. 13:**
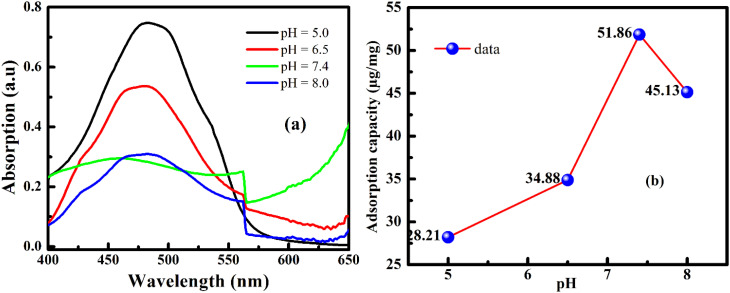
(a) UV-Vis absorption spectra of DOX during adsorption onto FG@SAP nanoparticles under different pH conditions, and (b) corresponding drug-loading capacity (DLC, µg mg^−1^) of FG@SAP as a function of pH (experimental details are provided in Table S2).

Fig. 13 shows the pH-dependent DOX adsorption on FG@SAP nanoparticles, along with the corresponding drug-loading capacity (µg mg^−1^). Experimental details and quantitative data are provided in Table S4. DOX adsorption is strongly influenced by solution pH, which governs electrostatic interactions and the ionization states of both DOX molecules and amino-functionalized surfaces.^48,49^

DOX contains amine and hydroxyl groups. At alkaline pH (>8), protonated amine groups (–NH_3_^+^) gradually deprotonate to –NH_2_, reducing aqueous solubility and enhancing hydrophobic interactions. Additionally, DOX tends to dimerize in alkaline conditions, further lowering effective solubility.^50^ These factors collectively reduce adsorption efficiency at high pH. In contrast, neutral pH (7.4) provides optimal conditions for DOX loading, maximizing electrostatic attraction and hydrogen bonding with surface –NH_2_ groups. Under these conditions, drug-loading efficiency reaches 82.6%, corresponding to a maximum capacity of 51.86 µg mg^−1^. Unmodified FG@S nanoparticles show a lower adsorption capacity (∼30 µg mg^−1^), highlighting the role of APTES functionalization in enhancing drug loading. For comparison, Hosseinzadeh *et al.*^51^ reported 27 mg g^−1^ DOX adsorption for Fe_3_O_4_@SiO_2_–NH_2_ nanoparticles with ∼74% release efficiency. Despite differences in experimental conditions, the FG@SAP system demonstrates competitive and superior loading efficiency, emphasizing the effectiveness of the tailored core–shell architecture and surface functionalization.

#### 3.3.2. Adsorption kinetics of DOX on FG@SAP nanoparticles

The adsorption of doxorubicin (DOX) onto APTES-modified FG@SAP nanoparticles was investigated using time-dependent UV-Vis spectroscopy combined with kinetic modeling. DOX concentrations at different adsorption times were determined from the UV-Vis spectra using the calibration curve in Fig. S3. The evolution of absorption spectra and corresponding drug-loading capacity (DLC, µg mg^−1^) is shown in Fig. S3, with detailed data in Table S5.

As adsorption proceeds, DOX absorbance decreases, indicating progressive uptake by FG@SAP nanoparticles. DLC increases rapidly during the initial 1–2 h, followed by a slower approach to equilibrium after ∼8 h. This suggests that initially abundant surface-active sites enable fast adsorption, which slows as sites become occupied and diffusion into less accessible regions becomes rate-limiting.

**Fig. 14:**
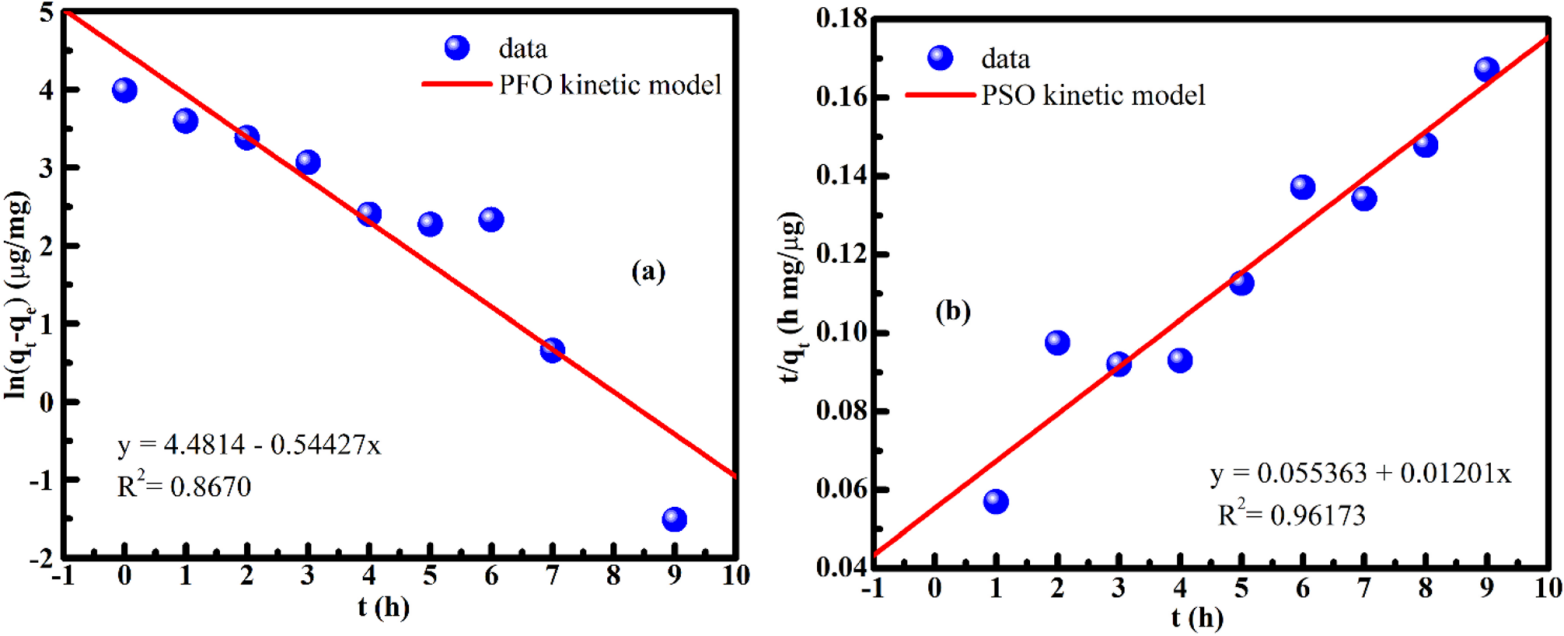
Kinetic modeling of doxorubicin (DOX) adsorption onto FG@SAP nanoparticles at pH 7.4 and 27 °C using (a) the pseudo-first-order (PFO) model and (b) the pseudo-second-order (PSO) model, indicating that the adsorption process is better described by the PSO model.

Time-dependent DLC data were fitted with pseudo-first-order (PFO) and pseudo-second-order (PSO) kinetic models^52^ (Fig. 14). The PSO model fits significantly better (*R*^2^ ≈ 0.97 *vs.* 0.76 for PFO), indicating chemisorption-dominated kinetics.

The strong agreement with the PSO model suggests specific interactions between surface amino groups of FG@SAP and DOX molecules *via* electrostatic attraction and hydrogen bonding. At pH 7.4, DOX is predominantly protonated, enabling interactions with amino-functionalized surfaces, including possible donor–acceptor interactions. These contribute to the high drug-loading efficiency and capacity observed.

Overall, DOX adsorption follows a two-stage mechanism: (i) rapid initial uptake driven by abundant surface-active sites and electrostatic/hydrogen-bond interactions, followed by (ii) slower approach to equilibrium controlled by surface saturation and diffusion-limited chemisorption. This mechanism aligns with the observed 8 h optimal adsorption time and supports the stability of the DOX-loaded nanocarrier, providing a solid basis for magnetically triggered drug release and *in vitro* therapeutic performance.

#### 3.3.3. pH-responsive DOX release behavior and release mechanism

The DOX release from FG@SAPD nanoparticles was evaluated at pH 4.2, 5.0, and 7.4 over 12 h (Fig. S4). Release profiles plateau after ∼6 h, with a maximum cumulative release of 60.4% at pH 4.2, demonstrating pronounced pH-responsive behavior. Enhanced release under acidic conditions is attributed to protonation-induced weakening of electrostatic and hydrogen-bonding interactions between DOX and the amino-functionalized surface, relevant to acidic tumor microenvironments.

**Fig. 15:**
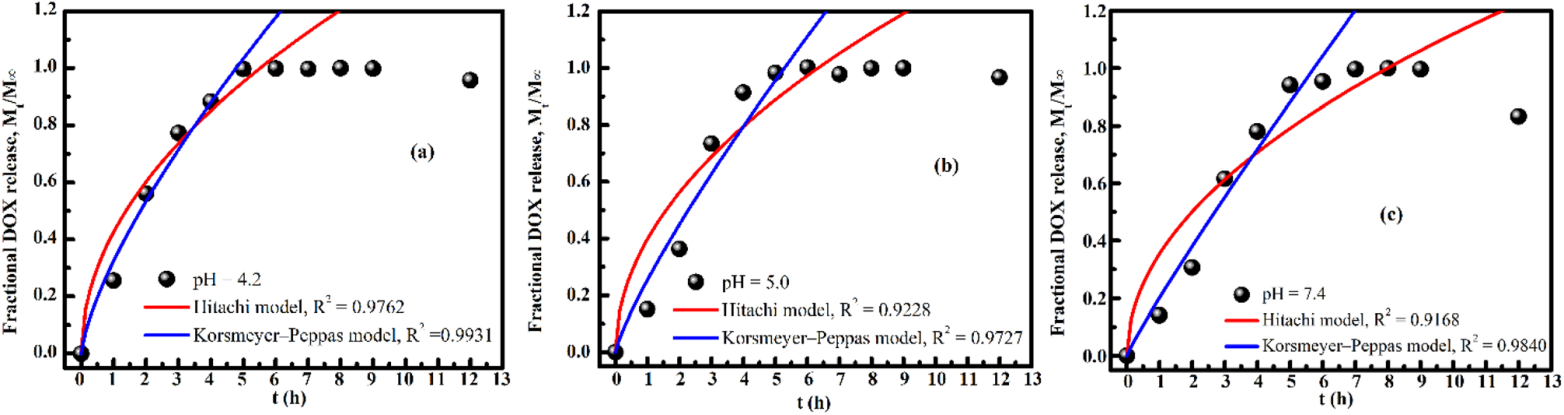
Fitting of cumulative doxorubicin (DOX) release data from FG@SAPD nanoparticles using the Higuchi and Korsmeyer–Peppas models under different pH conditions: (a) pH = 4.2, (b) pH = 5.0, and (c) pH = 7.4. The fitting was performed using release data collected within the first 6 h to elucidate the dominant drug-release mechanism.

To clarify the release mechanism, data from the first 6 h were fitted with Higuchi and Korsmeyer–Peppas models^53^ (Fig. 15). The Korsmeyer–Peppas model shows higher correlation (*R*^2^ = 0.9727–0.9930) than the Higuchi model, with a release exponent *n* = 0.72–0.92, indicating non-Fickian transport. This mechanism involves combined DOX diffusion through the porous SiO_2_ shell and relaxation of the APTES-modified surface.

Overall, the pH-responsive, diffusion–relaxation-controlled release behavior supports the potential of FG@SAPD nanoparticles for controlled and targeted anticancer drug delivery.

#### 3.3.4. AMF-assisted DOX release under alternating magnetic field

The AMF-responsive release of DOX from FG@SAPD nanoparticles was evaluated under an alternating magnetic field (250 Oe, 450 kHz). In a typical experiment, 30 mg of FG@SAPD was dispersed in 10 mL PBS (pH 7.4) and subjected to AMF exposure.

**Fig. 16:**
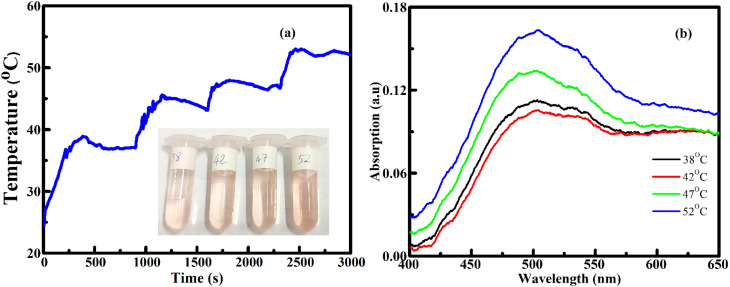
(a) AMF-induced magnetothermal heating curves of DOX-loaded FG@SAP nanoparticles at fixed temperatures of 38, 42, 47, and 52 °C with a holding time of 10 min (250 Oe, 450 kHz) and (b) UV-Vis absorption spectra of DOX released from FG@SAP nanoparticles after AMF treatment at different temperatures.

Fig. 16a shows the temperature evolution curves under AMF exposure, reaching bulk equilibrium temperatures of 38, 42, 47, and 52 °C within 10 min. The corresponding UV-Vis spectra of released DOX (Fig. 16b) were used to quantify cumulative release. A 10-min heating duration was selected to evaluate short-term, on-demand release behavior, since prolonged continuous AMF exposure over several hours is experimentally challenging. Within this interval, AMF stimulation resulted in higher DOX release compared to passive diffusion measured over the same duration. The cumulative release increased to 5.16% at 42 °C and 10.7% at 52 °C, whereas passive release over short timescales remained lower. For reference, comparable release levels under static conditions required substantially longer incubation (≈6 h), suggesting that elevated temperature promotes faster release within short time intervals.

The increase in DOX release correlates with the measured bulk temperature elevation of the suspension. Elevated temperature is expected to enhance molecular mobility within the APTES-modified silica shell and increase the effective diffusion of DOX, thereby facilitating its release from the nanocarrier matrix. Additional experiments involving continuous AMF-induced heating from 38 to 80 °C (Fig. S5 and Table S6) further show a progressive increase in cumulative release with temperature, supporting the temperature-dependent release behavior of the FG@SAPD system.

It should be noted that temperature monitoring reflects bulk suspension heating. Therefore, the observed release enhancement is interpreted primarily as a consequence of AMF-induced thermal effects. A direct comparison with conventional bulk heating (*e.g.*, water bath control) was not performed in the present study. Consequently, while the data demonstrate AMF-assisted enhancement of drug release associated with bulk heating, further studies are required to quantitatively distinguish purely thermal contributions from potential magnetically specific nanoscale effects.

### 3.4. *In vitro* cytotoxicity evaluation on HepG2 and MCF-7 cells

Baseline morphology of HepG2 and MCF-7 cells was examined prior to incubation. Untreated HepG2 cells exhibited typical adherent polygonal shapes, whereas MCF-7 cells displayed a flattened epithelial-like morphology with intact cell boundaries, indicating healthy states (Fig. S6a and b). After 48 h under control conditions (without DOX), both cell lines largely maintained their morphology, with no pronounced shrinkage or detachment (Fig. S6c and d), confirming that the incubation process itself did not induce cytotoxicity.

**Fig. 17:**
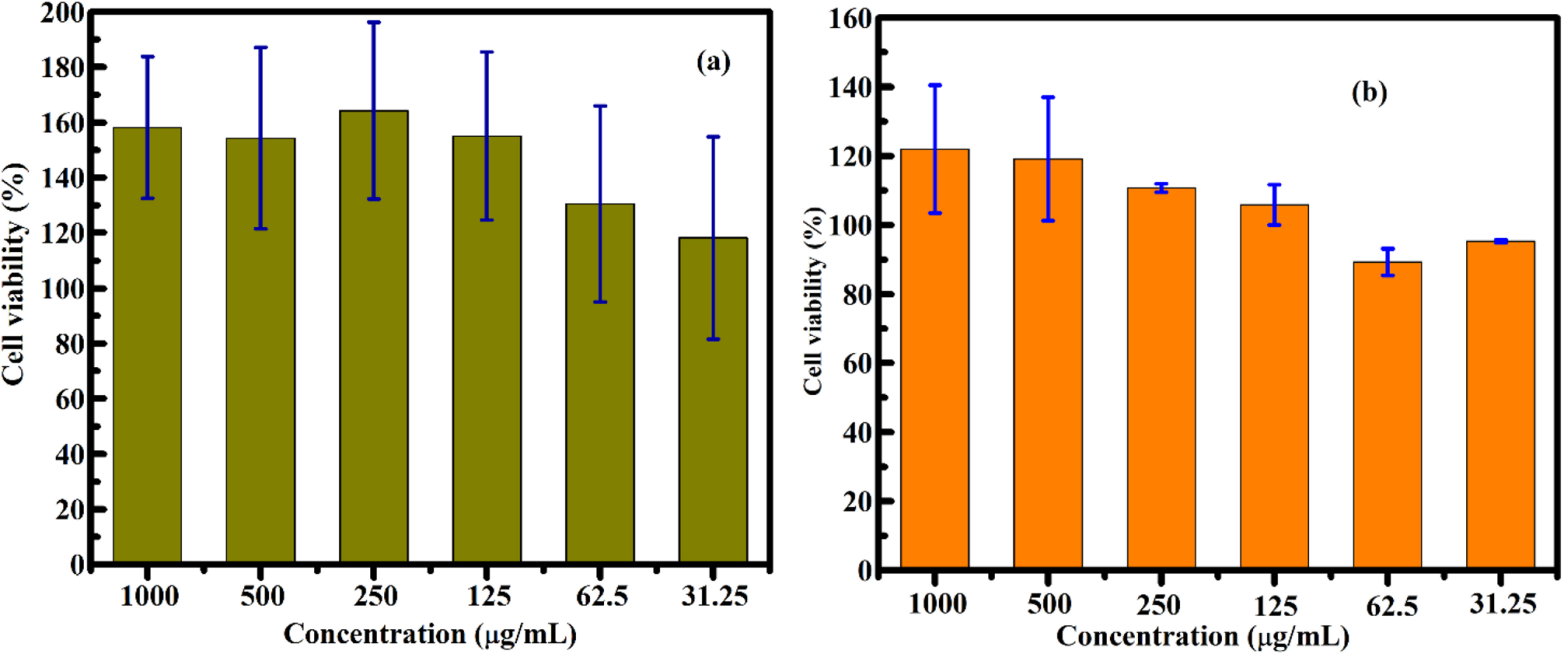
Cell viability of MCF-7 (a) and HepG2 (b) cells after 48 h incubation with the FG@S core–shell nanomaterial at different concentrations, evaluated using a colorimetric mitochondrial metabolic activity-based assay. Cell viability is expressed as a percentage relative to the untreated control cells.

Representative optical images of cells incubated with FG@S nanoparticles (250–1000 µg mL^−1^, 48 h) are shown in Fig. S7. Across all concentrations, FG@S did not induce apparent cytotoxic effects, as evidenced by preserved adhesion and high cell density. Even at 1000 µg mL^−1^, where a superficial nanomaterial layer formed on the cell surface, viable adherent cells remained underneath, demonstrating good *in vitro* biocompatibility. Quantitative assessment using a colorimetric mitochondrial activity assay (Fig. 17 and Table S7) confirmed high cell viability for both HepG2 and MCF-7 cells across all tested concentrations, with IC_50_ exceeding 1000 µg mL^−1^. Apparent viability values exceeding 100% reflect enhanced metabolic activity rather than prolifer ation.

For FG@SAPD, cytotoxicity was evaluated from 0.005 to 500 µg mL^−1^. Optical images at selected concentrations (500, 50, 5 µg mL^−1^) are shown in Fig. S8. At higher concentrations (50–500 µg mL^−1^), cells exhibited rounding, loss of adhesion, and reduced density, indicative of strong cytotoxicity. At 5 µg mL^−1^, partial recovery of morphology and adhesion was observed. Quantitative viability data (Table S8) show a concentration-dependent reduction in both cell lines.

Free DOX was evaluated from 0.002 to 200 µg mL^−1^ (Table S2 and Fig. S9). At 20–200 µg mL^−1^, severe morphological alterations and reduced cell density were observed, with effects persisting to ∼2 µg mL^−1^. Below 0.2 µg mL^−1^, partial recovery occurred, consistent with quantitative data (Table S9), confirming potent, concentration-dependent cytotoxicity.

**Fig. 18:**
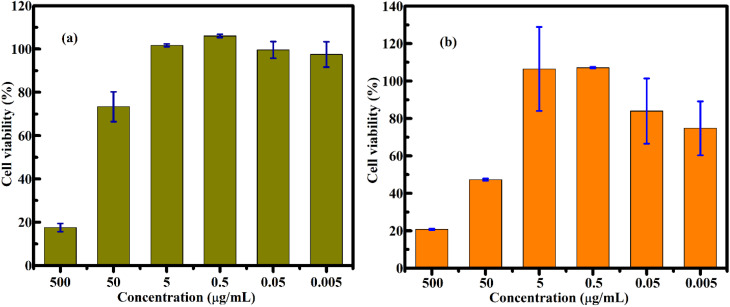
Cell viability of HepG2 (a) and MCF-7 (b) cells after 48 h incubation with the FG@SAPD core–shell nanocarrier at different concentrations, evaluated using a colorimetric mitochondrial metabolic activity-based assay. Cell viability is expressed as a percentage relative to untreated control cells.

**Fig. 19:**
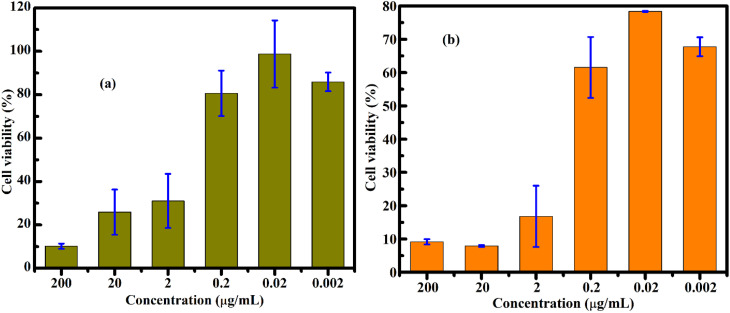
Cell viability of HepG2 (a) and MCF-7 (b) cells after 48 h incubation with free doxorubicin (DOX) at different concentrations, evaluated using a colorimetric mitochondrial metabolic activity-based assay. Cell viability is expressed as a percentage relative to untreated control cells.

Quantitative analysis of FG@SAPD and free DOX is shown in Fig. 18 and 19. FG@SAPD exhibited marked, concentration-dependent cytotoxicity, with cell viability falling below 50% at 50–500 µg mL^−1^. Free DOX showed stronger cytotoxicity, with viability <20% at ∼2 µg mL^−1^. IC_50_ values were 50.62 µg mL^−1^ (HepG2) and 47.60 µg mL^−1^ (MCF-7) for FG@SAPD, *versus* 0.60 µg mL^−1^ (HepG2) and 0.70 µg mL^−1^ (MCF-7) for free DOX. Considering the measured drug-loading capacity of 54.3 µg DOX per mg FG@SAPD, these correspond to effective DOX concentrations of ∼2.74 µg mL^−1^ (HepG2) and ∼2.58 µg mL^−1^ (MCF-7).

As summarized in Table S10, these DOX-equivalent IC_50_ values are comparable to other magnetic and silica-based DOX nanocarriers, confirming competitive anticancer efficacy. The moderated cytotoxicity of FG@SAPD relative to free DOX reflects controlled release and partial drug shielding, reducing nonspecific toxicity while maintaining therapeutic activity. Magnetic nanocarriers can enhance DOX efficacy *via* improved cellular uptake and intracellular accumulation,^54–57^ and the APTES-functionalized silica shell further contributes to biocompatibility, drug-loading efficiency, and sustained intracellular release.^58,59^

The present cytotoxicity evaluation was designed to examine the *in vitro* antiproliferative effect in representative malignant cell models. A comprehensive determination of selective cytotoxicity, however, requires systematic comparison with non-malignant cells to establish a quantitative therapeutic window. Such evaluation is particularly relevant for magnetothermally assisted systems, where differential sensitivity to thermal stress and drug exposure may influence overall cytotoxic selectivity. Future investigations should therefore incorporate normal epithelial or fibroblast cell lines under identical exposure conditions to further define biosafety margins and selective cytotoxicity profiles.

### 3.5. AMF-induced magnetothermal cytotoxicity in cancer cells

**Fig. 20:**
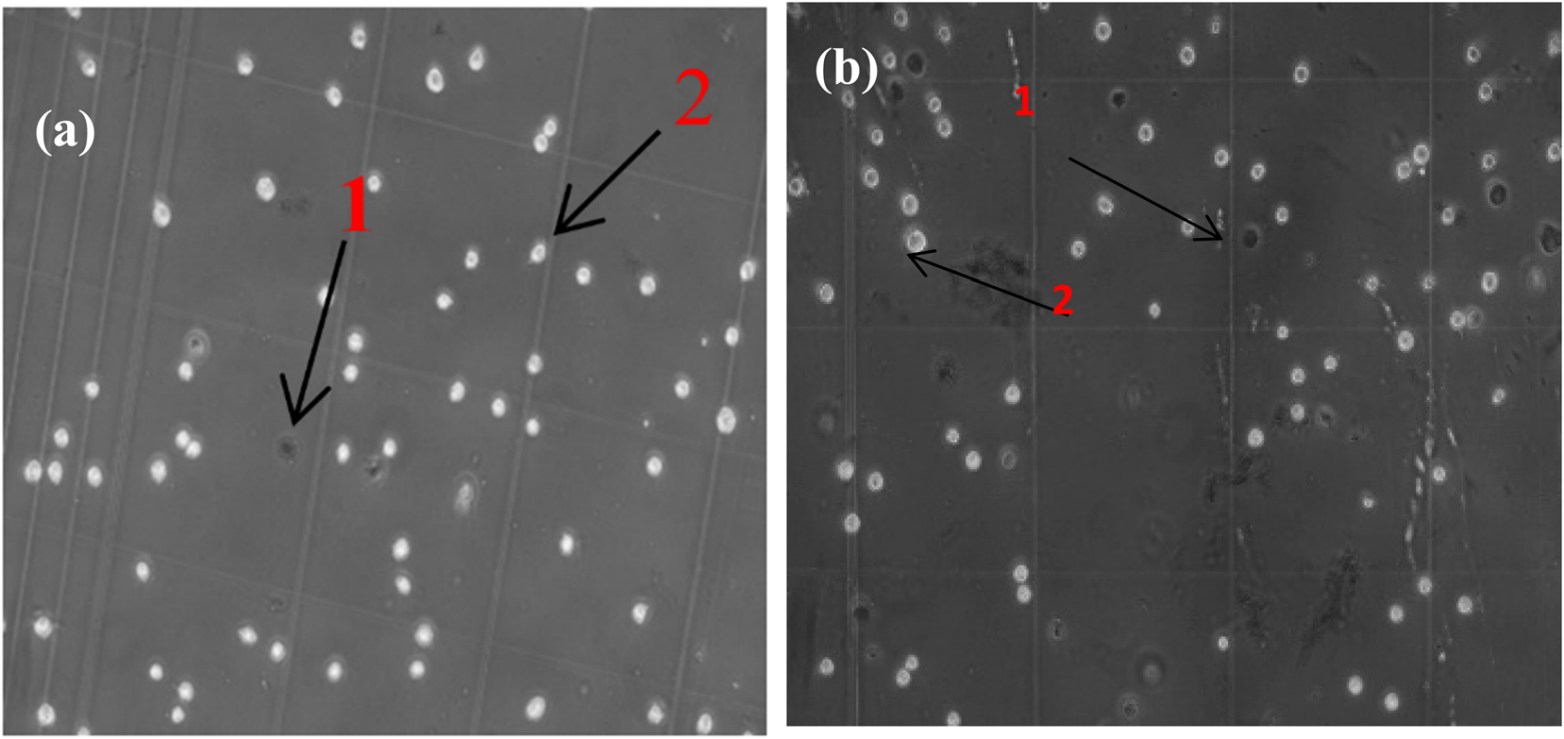
Representative optical microscopy images showing the morphology and live/dead cell ratio of MCF-7 cells in the control group (a) and in wells supplemented with drug-free FG@SAPD nanomaterial (b) (VK10 ×10 magnification). Dead cells are indicated by arrow (1), while viable cells are indicated by arrow (2), as identified by Trypan Blue staining.

To further evaluate baseline cytotoxicity prior to magnetic hyperthermia treatment, two control groups were established. As shown in Fig. 20, optical microscopy images were obtained for untreated cells and for cells incubated with FG@SAPD in the absence of an alternating magnetic field (AMF). Cell viability was quantified using the Trypan Blue exclusion assay. Both control groups maintained high viability (>95% for untreated cells and 87.1% for FG@SAPD-treated cells without AMF), indicating low intrinsic cytotoxicity of the nanocarrier under normothermic conditions.

**Fig. 21:**
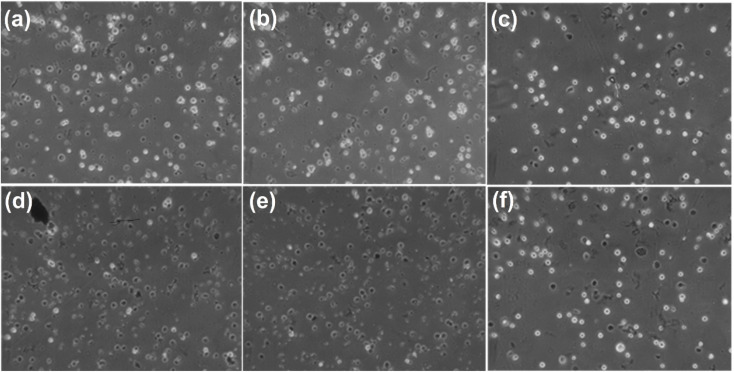
Representative optical microscopy images of MCF-7 cells illustrating morphological alterations following magnetothermal treatment of FG@SAPD core–shell DOX-loaded nanoparticles under an alternating magnetic field (VK10 ×10 magnification). Cells were exposed to AMF-induced heating at 45 °C for (a) 5 min and (b) 10 min, and at 55 °C for (d) 5 min and (e) 10 min. Control groups include cells incubated with FG@SAPD for (c) 5 min and (f) 10 min in the absence of AMF under normothermic conditions (37 °C).

In contrast, FG@SAPD-treated MCF-7 cells exposed to AMF exhibited temperature- and time-dependent reductions in cell viability, accompanied by evident morphological alterations (Fig. 21). At 55 °C, cell death exceeded 80% after 5 min and 90% after 10 min of exposure. These results demonstrate that magnetothermal heating generated under AMF can effectively induce substantial cytotoxicity *in vitro*, with the magnitude of the effect correlating with the achieved bulk temperature and exposure duration.

### 3.6. Comparative analysis with reported Fe_3_O_4_@SiO_2_-DOX systems

To position the present platform relative to representative magnetic silica-based DOX nanocarriers reported in the literature, a comparison of key functional parameters is summarized in Table S10. The drug-loading capacity of 54.3 µg mg^−1^ and loading efficiency of 85.3% place the system within the higher range of values reported for comparable Fe_3_O_4_@SiO_2_-based carriers.

Under moderate AMF parameters (200 Oe, 450 kHz), the measured SAR value of 153 W g^−1^ at 1 mg mL^−1^ indicates efficient magnetothermal conversion without the need for excessively high field amplitudes. This performance falls within the range reported for silica-coated magnetic systems operating under comparable excitation conditions.

The DOX-equivalent IC_50_ values (∼2.6–2.7 µg mL^−1^) are consistent with previously reported magnetically assisted DOX delivery systems, indicating comparable *in vitro* anticancer activity. Importantly, unlike conventional diffusion-controlled Fe_3_O_4_@SiO_2_-DOX platforms, the present system integrates pH-sensitive release with externally regulated AMF-induced thermal activation, providing the potential for dual-mode modulation of drug release through pH responsiveness and AMF-induced heating.

## 4. Conclusion

In this study, APTES-functionalized Gd_0.18_Fe_2.82_O_4_@SiO_2_ core–shell nanoparticles were successfully synthesized and evaluated as a magnetically activatable doxorubicin (DOX) nanocarrier exhibiting combined pH-responsive and AMF-regulated release behavior. Structural and morphological analyses confirmed quasi-spherical nanoparticles with well-defined magnetic cores and mesoporous silica shells, while DLS and zeta potential measurements demonstrated satisfactory colloidal stability in physiological-mimicking media. FTIR analysis indicated effective DOX adsorption mediated by electrostatic interactions and hydrogen bonding with the amino-functionalized surface.

The system achieved a high drug-loading efficiency (up to 82.6%), with adsorption kinetics consistent with a pseudo-second-order model. *In vitro* release studies revealed pronounced pH-dependent behavior, with accelerated release under acidic conditions. Upon exposure to an alternating magnetic field, bulk magnetothermal heating of the suspension was induced, leading to rapid, externally regulated enhancement of DOX release. The correlation between temperature elevation and cytotoxic response suggests that thermal stress plays a primary role in the observed AMF-assisted cytotoxic activation.

Cytotoxicity assays demonstrated negligible intrinsic toxicity of the nanocarrier in the absence of stimulation, whereas the DOX-loaded system induced concentration-dependent antiproliferative effects in HepG2 and MCF-7 cells. Under AMF exposure, substantial loss of cell viability (>90% at 55 °C within 10 min) was observed, demonstrating pronounced magnetothermally assisted cytotoxicity under controlled *in vitro* conditions.

Collectively, the Gd_0.18_Fe_2.82_O_4_@SiO_2_/APTES/DOX platform integrates high drug-loading capacity with pH-dependent release and AMF-induced thermal modulation. By combining these features, the system represents a dual-responsive chemo-thermal platform with potential for externally guided therapeutic applications. Further studies are required to quantitatively establish therapeutic selectivity and biosafety margins under physiologically relevant conditions.

## Author contributions

Pham Hoai Linh: writing – original draft. Tran Thi Huong: validation, methodology, investigation, formal analysis, conceptualization, data curation, Nguyen Hong Nhung: synthesis samples, validation, investigation, formal analysis, conceptualization. Tran Thi Ngoc Nha: writing – original draft, formal analysis, Pham Thanh Phong: conceptualization, investigation, formal analysis, data curation, writing – review & editing.

## Conflicts of interest

The authors declare that they have no conflict of interest.

## Supplementary Material

RA-016-D6RA00215C-s001

## Data Availability

All data generated or analyzed during this study are included in this published article and its supplementary information (SI) files. Supplementary information: contains additional experimental details, including procedures for determining drug-loading efficiency, drug-release capacity and DOX calibration, supplementary UV–Vis spectra, temperature-dependent magnetothermal drug-release analyses, optical microscopy images of HepG2 and MCF-7 cells under different treatments, quantitative cell-viability datasets, and a comparative overview of representative Fe₃O₄@SiO₂-based magnetic nanocarriers reported in the literature. See DOI: https://doi.org/10.1039/d6ra00215c.
