# Supplementary material for: APTES-functionalized Gd0.18Fe2.82O4@SiO2 nanocarrier for magnetothermal-triggered doxorubicin release
Source: RSC Adv. 2026 Mar 11;16(15):13708–27. doi: 10.1039/d6ra00215c (PMC12977968; doi:10.1039/d6ra00215c)
Supplement: RA-016-D6RA00215C-s001 [file RA-016-D6RA00215C-s001.pdf]

Supplementary Information (SI) for RSC Advances.
This journal is © The Royal Society of Chemistry 2026

Supporting Information

APTES-Functionalized $Gd_{0.18}Fe_{2.82}O_4@SiO_2$ Nanocarrier for Magnetothermal-Triggered Doxorubicin Release

Pham Hoai Linh[1], Tran Thi Huong[1], Nguyen Hong Nhung[1], Tran Thi Ngoc Nha[2], Pham Thanh Phong[3, 4]

[1]*Institute of Materials Science, Vietnam Academy of Science and Technology, 18-Hoang Quoc Viet, Hanoi City, Viet Nam*

[2]*Graduate University of Science and Technology, Vietnam Academy of Science and Technology, 18-Hoang Quoc Viet, Hanoi City, Vietnam*

[3]*Laboratory of Magnetism and Magnetic Materials, Science and Technology Advanced Institute, Van Lang University, Ho Chi Minh City, Viet Nam*

[4]*Faculty of Applied Technology, School of Technology, Van Lang University, Ho Chi Minh City, Viet Nam*

Supporting Information for Section 2.4: Preparation of DOX-loaded nanoparticles

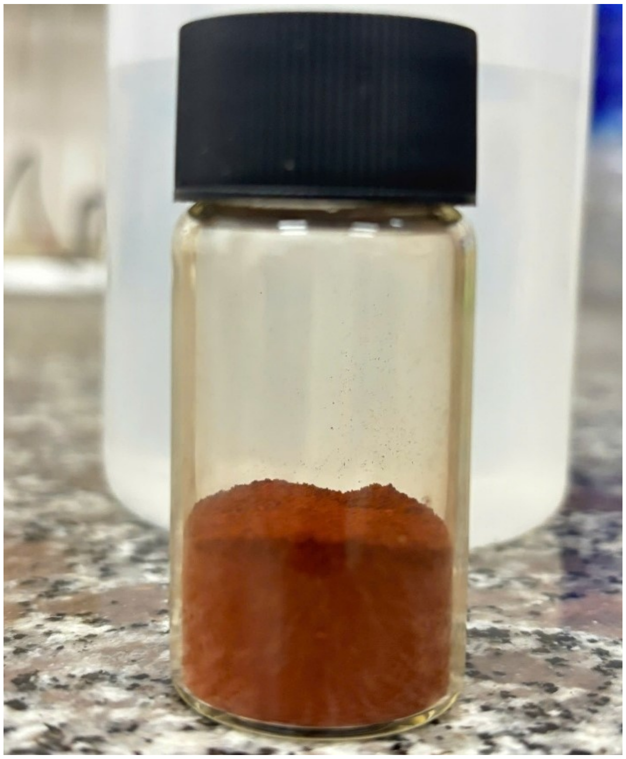

Fig. S1. Photograph of the freeze-dried $Gd_{0.18}Fe_{2.82}O_4@SiO_2$/APTES/DOX nanoparticles (FG@SAPD) obtained after lyophilization, showing a homogeneous reddish-brown powder with good visual uniformity.

Supporting Information for Sec

tion 2.6: Quantitative Analysis of DOX Adsorption and Release

This Supporting Information provides detailed quantitative methods used for evaluating doxorubicin (DOX) adsorption and magnetically triggered desorption experiments described in Section 2.6 of the main text, including the definitions of drug-loading efficiency (DLE), drug-loading capacity (DLC), drug release capacity (DRC), and the construction of the DOX calibration curve.

S2.6.1. Determination of Drug-Loading Efficiency (DLE) and Drug-Loading Capacity (DLC)

Drug adsorption experiments were conducted to evaluate the doxorubicin (DOX) loading performance of the FG@SAP nanocarriers. Briefly, DOX was dissolved in phosphate-buffered saline (PBS, pH 7.4) to obtain a stock solution with a concentration of 200 ppm. A known mass of FG@SAP nanoparticles (30 mg) was accurately weighed and dispersed in 10 mL of the DOX solution. The suspension was shaken continuously in the dark at room temperature for up to 12 h to reach adsorption equilibrium.

At predetermined time intervals, 1 mL aliquots were withdrawn and centrifuged to separate the nanoparticles. The supernatant was analyzed by UV–Vis spectroscopy at 483 nm to determine the residual DOX concentration. The drug-loading efficiency (DLE) and drug-loading capacity (DLC) were calculated according to the following equations:

$$DLE(\%) = \frac{C_t}{C_0} \times 100\% \qquad \text{(S1)}$$

$$DLC(\%) = \frac{m_t}{m_0} \times 100\% \qquad \text{(S2)}$$

where C_0 is the initial DOX concentration (200 ppm), $C_t = C_0 - C_R$ is the amount of DOX adsorbed by the nanoparticles, C_R is the residual DOX concentration in solution after adsorption, m_t(mg) is the mass of DOX loaded onto the nanoparticles, and m_0(mg) is the initial mass of the nanoparticle carrier used in the experiment.

S2.6.2. Determination of Drug Release Capacity (DRC)

The magnetically triggered release behavior of DOX-loaded nanoparticles was investigated using a dialysis method. Initially, 30 mg of FG@SAP nanoparticles were saturated with DOX by dispersing them in 10 mL of a 200 ppm DOX solution (PBS, pH 7.4) and shaking for 8 h. The

DOX-loaded nanoparticles were then collected by centrifugation and gently rinsed with distilled water to remove physically adsorbed DOX.

The resulting DOX-loaded nanoparticles were transferred into a dialysis bag and immersed in 10 mL of fresh PBS solution. The system was maintained at 42 °C under continuous shaking. For magnetically triggered release experiments, the sample was exposed to an alternating magnetic field (AMF) with a field strength of 250 Oe and a frequency of 450 kHz for a specified duration, as described in the main text.

At selected time intervals, 1 mL of the release medium was withdrawn and analyzed by UV–Vis spectroscopy at 483 nm to quantify the released DOX, after which an equal volume of fresh PBS was replenished to maintain a constant release volume. The drug release capacity (DRC) was calculated using the following expression:

$$DRC(\%) = \frac{C_t}{C_L} \times 100\% \quad \text{(S3)}$$

where C_t is the DOX concentration in the release medium at time t, and C_L is the total DOX concentration initially loaded onto the nanoparticles.

S2.6.3. Construction of the DOX Calibration Curve

The calibration curve for doxorubicin quantification was established using ultraviolet–

Table S1. UV–Vis absorbance values of doxorubicin (DOX) aqueous solutions at different concentrations measured at 483 nm.

Concentration (ppm)	1	2.5	5	7	10	15	20	31	63	125
Absorbance (a.u)	0.088	0.116	0.162	0.245	0.251	0.341	0.435	0.66	1.183	2.35

visible (UV–Vis) absorption spectroscopy. A series of standard DOX solutions with concentrations of 1, 2.5, 5, 7.5, 10, 15, 31, 62, and 125 ppm were prepared in phosphate-buffered saline (PBS, pH 7.4). DOX exhibits a characteristic absorption maximum at 483 nm; therefore, all measurements were performed at this wavelength using a 1 cm path-length quartz cuvette.

The absorbance values corresponding to each DOX concentration (**Tab. S1**) were used to construct the calibration curve (**Fig. S2**). A clear linear relationship between absorbance and DOX concentration was obtained over the investigated range of 1–125 ppm, with a correlation coefficient of $R^2 > 0.9992$. This high degree of linearity confirms that the selected concentration range lies within the Beer–Lambert regime and validates the use of the calibration curve for accurate quantification of DOX during both adsorption and magnetically triggered release experiments.

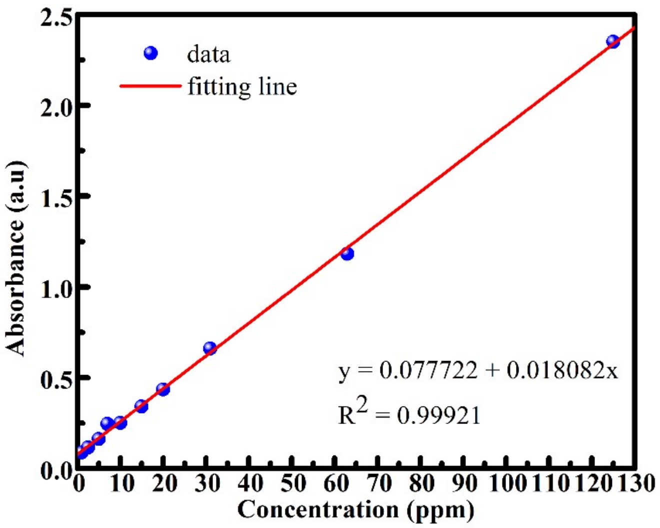

Figure S2. UV–Vis calibration curve of doxorubicin (DOX) constructed from absorbance measured at 483 nm over the concentration range of 1–125 ppm, showing excellent linearity ($R^2 > 0.9992$).

Supporting Information for Section 2.7: Cytotoxicity Assay and Cellular Studies

This section provides detailed experimental procedures and supplementary data for the in vitro cytotoxicity evaluation and cellular studies described in Section 2.7 of the main text. The

Tab. S2. Concentration ranges of FG@SAPD, free DOX, and solvent control used for in vitro cytotoxicity evaluation

Group	Samples	Testing concentration [C] (μg/mL)					
		[C1]	[C2]	[C3]	[C4]	[C5]	[C6]
Material control	FG@S	1000	500	250	125	62.5	31.25
Drug carrier	FG@SAPD	500	50	5	2.5	1.25	0.625
Positive control	Doxorubicin	200	2	0.2	0.002	0.0002	0.00002
Negative control	PBS (1X)	10%	10%	10%	10%	10%	10%

analyses include:

Note: PBS (1X, pH 7.4) was employed as the vehicle control, as it does not affect cell viability under the experimental conditions.

Supporting Information for Section 2.8: In Vitro Magnetothermal Treatment of MCF-7 Cells

This section provides detailed experimental procedures and supplementary data for the magnetothermal treatment of MCF-7 cells described in Section 2.8 of the main text. The analyses include:

Tab. S3. Experimental groups used to evaluate the in vitro magnetothermal therapeutic effect of DOX-loaded FG@SAP nanoparticles in MCF-7 cells. Cells were treated with FG@SAPD at a concentration corresponding to half of the IC_{50} value (25 µg mL^{-1}) and exposed to an alternating magnetic field (AMF) for different durations. Control groups with phosphate-buffered saline (PBS, 1X) and without AMF exposure were included to distinguish intrinsic cytotoxicity from magnetothermal-induced effects. The reported temperatures correspond to the bulk temperatures reached during AMF exposure.

Group	Samples	Concentration (µg/mL)	AMF exposure	Duration	Observed temp.
G1	PBS (1X)	-	No	-	37°C
G2	FG@SAPD	25 (½IC_{50})	No	-	37°C
G3	PBS (1X)	-	Yes	5 min	~45°C
G4	PBS (1X)	-	Yes	10 min	~55°C
G5	FG@SAPD	25 (½IC_{50})	Yes	5 min	~45°C
G6	FG@SAPD	25 (½IC_{50})	Yes	10 min	~55°C

Supporting Information for Section 3.3: Adsorption and magnetically triggered desorption of DOX

This section provides detailed experimental procedures, supplementary data, and quantitative analyses for the adsorption of doxorubicin (DOX) onto FG@SAP nanoparticles and magnetically triggered desorption as described in Section 3.3 of the main text. Considering the high thermal diffusivity of aqueous media, heat generated at the nanoparticle surface is expected to dissipate rapidly into the surrounding solution, leading to near-equilibrium bulk heating conditions. Therefore, the biological and release effects observed in this work are interpreted within the framework of bulk thermal activation rather than persistent nanoscale temperature gradients. The following content is included:

S3.3.1. DOX Adsorption Behavior under Static Conditions

For pH-dependent adsorption studies, 30 mg of the GF@SAP nanoparticles were dispersed in 10 mL of doxorubicin (DOX) aqueous solution (200 ppm) prepared in buffer solutions with pH

Table S4. Drug-loading efficiency (DLE, %), drug-loading capacity expressed as mass ratio (DLC, %), and drug-loading capacity expressed as DOX amount per unit mass of nanoparticles (DLC, μg mg^{-1}) of FG@SAP at different pH values. For convenience, DLC is reported both as a percentage and as the amount of DOX loaded per unit mass of nanoparticles (μg mg^{-1})

pH	Drug-loading efficiency (DLE, %)	Drug-loading capacity (DLC, %)	Drug-loading capacity (DLC, μg mg^{-1})
5.0	50.55	2.84	28.21
6.5	68.79	3.53	34.88
**7.4**	**82.56**	**5.18**	**51.86**
8.0	84.96	4.56	45.13

values of 5.0, 6.5, 7.4, and 8.0. The suspensions were magnetically stirred at room temperature (25 °C) for 7 h to reach adsorption equilibrium. After adsorption, the nanoparticles were separated by centrifugation, and the residual DOX concentration in the supernatant was quantified by UV–Vis spectroscopy at 483 nm. The corresponding drug loading efficiency (DLE) and drug loading capacity (DLC) are summarized in **Tab. S4**

S3.3.2. Adsorption kinetics of DOX on FG@SAP nanoparticles

Table S5. Time-dependent drug-loading efficiency (DLE, %), drug-loading capacity (DLC, %), and drug-loading capacity expressed as mass loading (DLC, μg mg^{-1}) of FG@SAP nanoparticles during DOX adsorption at pH 7.4. For convenience, DLC is reported both as a percentage and as the amount of DOX loaded per unit mass of nanoparticles (μg mg^{-1})

Time (h)	Drug-loading efficiency (DLE, %)	Drug-loading capacity (DLC, %)	Drug-loading capacity (DLC, μg mg^{-1})
0	0	0	0
1	27.81	1.76	17.56
2	32.47	4.10	20.50
3	51.66	6.53	32.61
4	68.11	8.60	43.00
5	70.30	8.88	44.38
6	69.29	8.75	43.74
7	82.60	10.44	52.14
8	85.66	10.82	54.07
9	85.30	10.78	53.85

Sixty milligrams of the magnetic nanoparticle sample (FG@SAP) were dispersed in 20 mL of a 200 ppm DOX solution (pH = 7.4) and allowed to adsorb at room temperature (27 °C). The adsorption process was monitored over a period of 1–9 h.

A rapid increase in DOX uptake is observed during the initial adsorption period, followed by a more gradual increase with prolonged contact time. The drug-loading efficiency exceeds 85% after 8 h, indicating that adsorption equilibrium is nearly reached. A slight decrease in loading parameters at longer times suggests the establishment of an adsorption–desorption equilibrium. Based on these results, an adsorption time of 8 h was selected as the optimal condition for subsequent experiments.

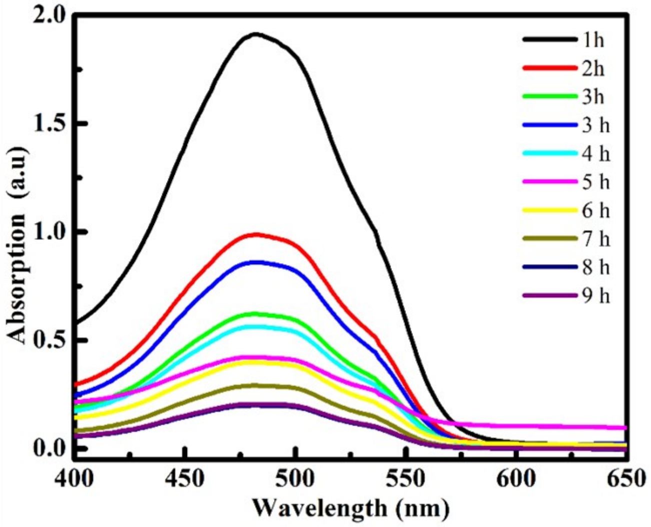

Figure S3. UV-Vis absorption spectra and corresponding time-dependent drug-loading capacity (DLC, μg mg^{-1}) of FG@SAP nanoparticles during doxorubicin (DOX) adsorption at pH 7.4 and 27 °C

Fig. S3 shows the UV–Vis absorption spectra and the corresponding DOX loading capacity (μg mg^{-1}) of the FG@SAP sample as a function of adsorption time. The quantitative values of drug-loading efficiency (DLE, %), drug-loading capacity (DLC, %), and drug-loading capacity expressed in mass units (μg mg^{-1}) are summarized in **Tab. S5**.

S3.3.3. pH-Responsive DOX Release Behavior and Release Mechanism

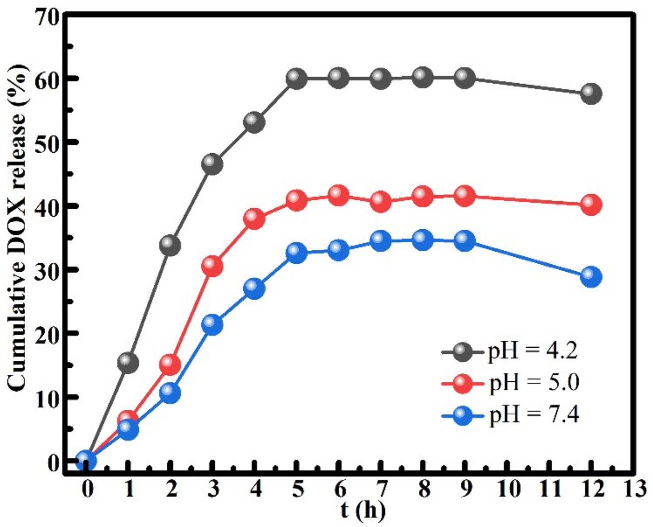

Figure S4. Time-dependent cumulative DOX release (%) from FG@SAPD nanoparticles under different pH conditions, used for kinetic modeling of the release process.

S3.3.4. AMF-assisted DOX release under alternating magnetic field

In this experiment, 30 mg of the FG@SAPD material sample was dispersed in 10 mL of PBS buffer solution (pH = 7.4) and subjected to continuous heating under an alternating magnetic field.

Table S6. Temperature-dependent drug-release capacity of FG@SAPD under continuous magnetic heating

Continuous heating temperature (°C)	38	42	47	52	57	62	67	72	80
Drug-release capacity (%)	4.2	4.1	4.3	4.1	4.5	5.6	5.5	6.8	10.6

During the heating process, 0.5 mL aliquots of the supernatant were collected at predefined temperatures of 38, 42, 47, 52, 57, 63, 67, 72, and 80 °C for UV–Vis analysis of the released DOX.

Tab. S6 summarizes the temperature-dependent drug-release capacity of the FG@SAPD nanocarrier system under continuous AMF-induced heating. In the temperature range of 38–52 °C, the cumulative drug release remains nearly constant (~4.1–4.3%), indicating good thermal stability

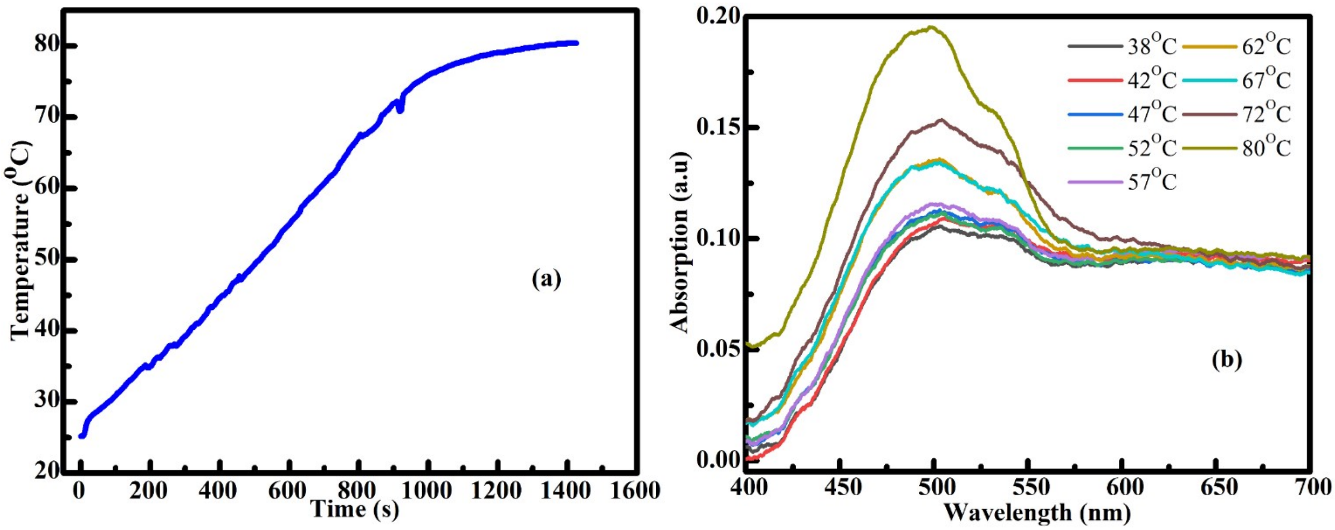

Fig. S5. Temperature-dependent drug-release analysis of FG@SAPD under continuous magnetic heating (a) Time evolution of the sample temperature during continuous AMF-induced heating from 25 to 80 °C. The cumulative drug-release values were recorded at selected temperatures corresponding to those listed in Tab.S6. (b) UV–Vis absorption spectra of the released DOX collected at the corresponding temperatures during continuous magnetic heating, from which the cumulative drug-release values summarized in Tab. S6 were extracted.

and negligible premature leakage under physiologically relevant conditions. When the temperature exceeds 57 °C, a progressive increase in drug release is observed, reaching a maximum value of 10.6% at 80 °C. This behavior further supports the thermally responsive release mechanism of the FG@SAPD system under magnetic heating, while the high-temperature data are intended to qualitatively validate the thermal activation effect rather than to represent physiological operating conditions.

Supporting Information for Section 3.4. In vitro cytotoxicity evaluation on HepG2 and MCF-7 cells

.

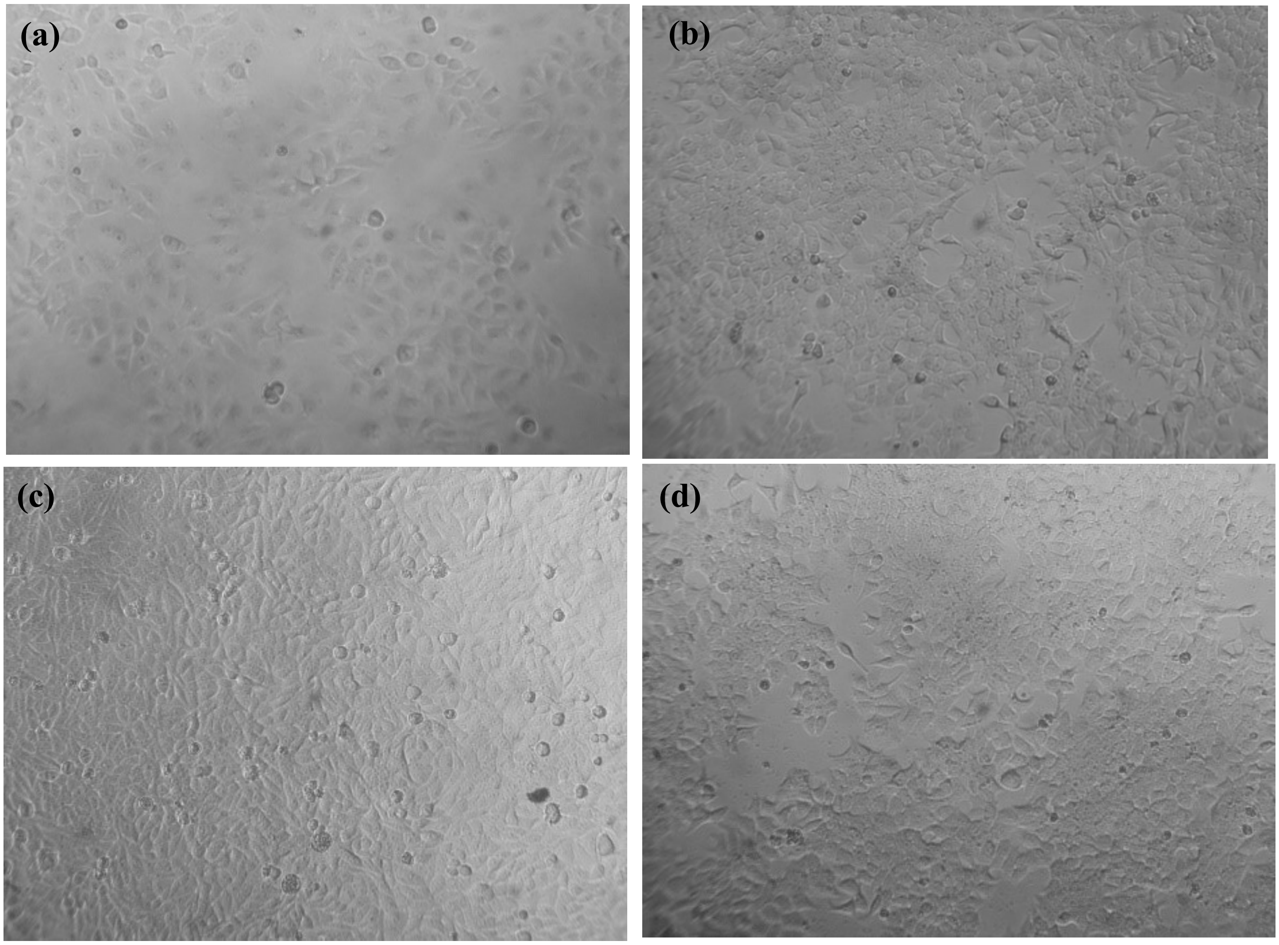

Fig. S6. Optical microscopy images of HepG2 and MCF-7 cells before incubation (0 h) ((a) HepG2, (b) MCF-7) and after 48 h incubation under control conditions (without DOX) ((c) HepG2, (d) MCF-7)

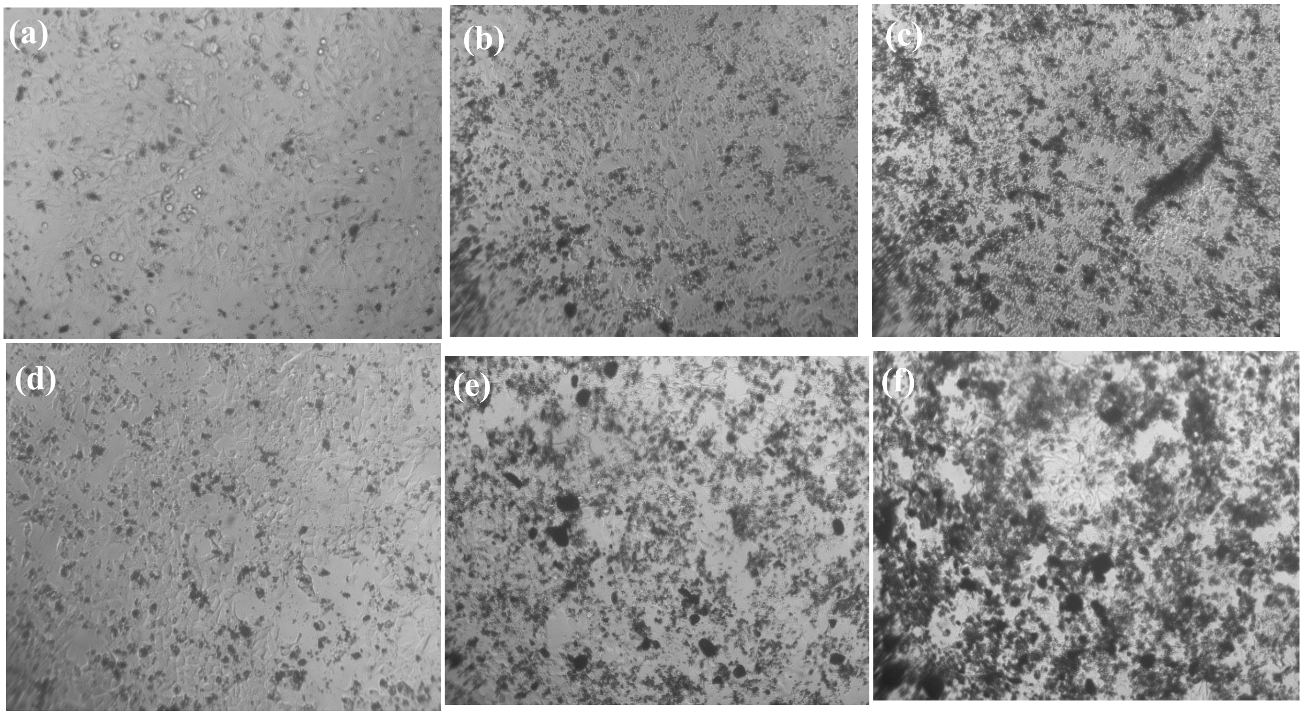

Fig. S7. Optical microscopy images of HepG2 and MCF-7 cancer cells after 48 h incubation with the FG@S core–shell nanomaterial at different concentrations. Panels (a–c) show HepG2 cells treated with FG@S at 250 (a), 500 (b), and 1000 µg/mL (c), while panels (d–f) correspond to MCF-7 cells treated at 250 (d), 500 (e), and 1000 µg/mL (f).

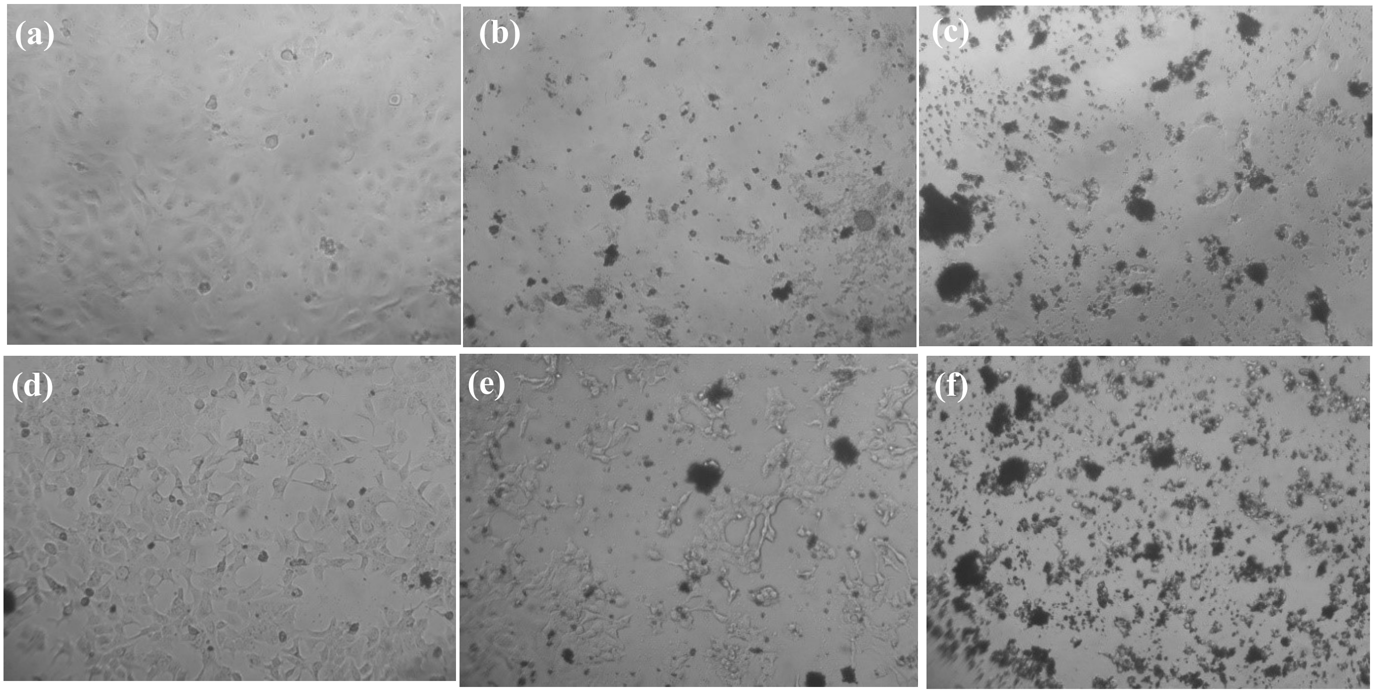

Fig. S8. Optical microscopy images of HepG2 and MCF-7 cancer cells after 48 h incubation with the FG@SAPD nanomaterial at different concentrations. Panels (a–c) show HepG2 cells treated with FG@S at 5 (a), 50 (b), and 500 µg/mL (c), while panels (d–f) correspond to MCF-7 cells treated at 5 (d), 50 (e), and 500 µg/mL (f).

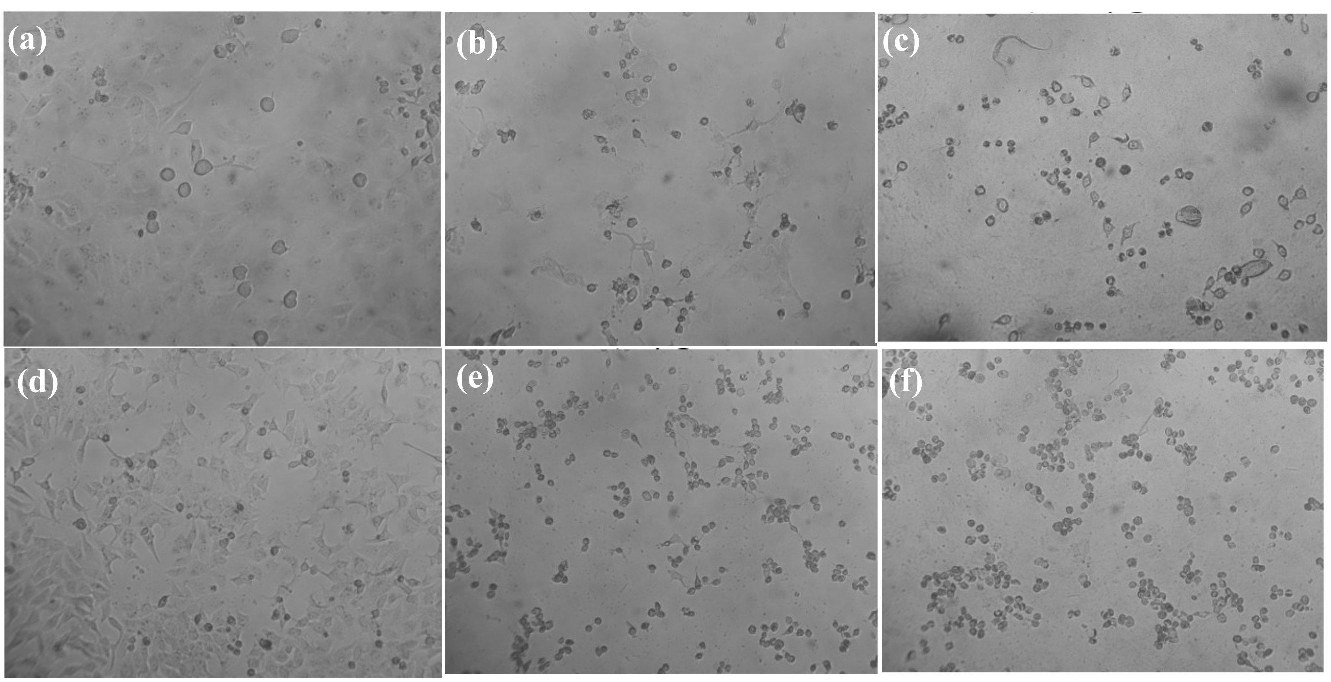

Fig. S9. Optical microscopy images of HepG2 and MCF-7 cancer cells after 48 h incubation with the doxorubicin at different concentrations. Panels (a–c) show HepG2 cells treated with FG@S at 0.2 (a), 2 (b), and 200 µg/mL (c), while panels (d–f) correspond to MCF-7 cells treated at 0.2 (d), 2 (e), and 200 µg/mL (f).

Table S7. Quantitative cell viability data of MCF-7 and HepG2 cells after 48 h incubation with the FG@S core–shell nanomaterial at different concentrations. Data are presented as mean ± SD (*n* = 3).

Cell	Concentration (µg/mL)	Cell viability (%)	SD (%)	Cell	Concentration (µg/mL)	Cell viability (%)	SD (%)
MCF-7	1000	158.14	25.63	HepG2	1000	121.97	18.51
	500	154.25	32.81		500	119.11	17.90
	250	164.26	32.04		250	110.70	1.21
	125	155.05	30.44		125	105.85	5.85
	62.5	130.48	35.49		62.5	89.29	3.84
	31.25	118.18	36.62		31.25	95.25	0.43

Table S8. Quantitative cell viability data of MCF-7 and HepG2 cells after 48 h incubation with the FG@SAPD nanomaterial at different concentrations. Data are presented as mean ± SD (*n* = 3).

Cell	Concentration (μg/mL)	Cell viability (%)	SD (%)	Cell	Concentration (μg/mL)	Cell viability (%)	SD (%)
MCF-7	500	17.50	1.91	HepG2	500	20.73	0.37
	50	73.35	6.87		50	47.29	0.57
	5	101.71	0.64		5	106.46	22.44
	0.5	106.03	0.76		0.5	107.14	0.43
	0.05	99.62	3.88		0.05	83.94	17.41
	0.005	97.48	5.89		0.005	74.75	14.40

Table S9. Quantitative cell viability data of MCF-7 and HepG2 cells after 48 h incubation with the doxorubicin at different concentrations. Data are presented as mean ± SD (n = 3).

Cell	Concentration (μg/mL)	Cell viability (%)	SD (%)	Cell	Concentration (μg/mL)	Cell viability (%)	SD (%)
MCF-7	200	10.19	1.18	HepG2	200	9.18	0.77
	20	25.88	10.40		20	7.93	0.31
	2	31.05	12.45		2	16.80	9.17
	0.2	80.62	10.45		0.2	61.54	9.15
	0.02	98.76	15.49		0.02	78.38	0.15
	0.002	85.94	4.29		0.002	67.76	2.84

Supporting Information for Section 3.4. Comparative analysis with reported Fe_3O_4@SiO_2-DOX systems

Table S10. Quantitative comparison of representative $Fe_3O_4@SiO_2$-Dox-based magnetic nanocarriers reported in the literature.

Nanocarrier system	Cell line	Drug-loading efficiency (DLE, %)	Cumulative release (%) at pH 7.4	IC_{50} (µg/mL, DOX-equivalent)	SAR (Wg^{-1})	AMF condition (Oe/kHz)	Stimulus/ release mechanism	Ref.
FG@SAPD	HepG2	**51.86**	**34% (8 hours)**	**≈ 2.74**	**153**	**200/450**	AMF-induced magnetothermal + thermal diffusion	This work
	MCF-7			**≈ 2.58**			AMF-induced magnetothermal + thermal diffusion	This work
Chitosan/ Fe_3O_4/SiO_2-DOX	HepG2	-	50% (24 hours)	2.65	-	-	pH-responsive	[1]
Chitosan/ Fe_3O_4/SiO_2-DOX	MCF-7			2.25			pH-responsive	[1]
$Fe_3O_4@SiO_2$/ PEG- DOX	HepG2			5.17	16.7	270/230	pH-responsive	[2]
Fe_3O_4@FA-PEI-SUC-DOX	MCF-7	-	2% (1 hours)	45.7			pH-responsive	[3]
Fe_3O_4@P (MEO_2MA_{60} −$OEGMA_{40}$) -DOX	SKOV-3	56.1	20% (25 hours)	0.07	25.2	300/536.5	AMF-induced magnetothermal + thermal diffusion	[4]
Fe_3O_4NPs@ SiO_2-g-poly(NVCL-co-DEAEMA-DOX	Human blood (healthy donors)	34.5	35% (10 hours)	-	-	-	pH-responsive	[5]
$Fe_3O_4@SiO_2$ -Folic acid-chitosan	Hela cell	36.1	18% (pH=7.4, 24 hours)	1.82 µg/mL	-	-	AMF-induced magnetothermal + thermal diffusion	[6]
Fe_3O_4-MSN-TESPA-PEG/DOX	N/A	-	74.02% (pH=7.4, 12 hours)		111.10	500/300	Photothermal + thermal diffusion	[7]

Note: IC_{50} values are normalized to the effective doxorubicin concentration to ensure fair comparison across different carrier systems. For FG@SAPD, DOX-equivalent IC_{50} values were calculated based on a drug loading capacity of 54.3 μg DOX per mg carrier. Data for literature systems were extracted directly from the corresponding references.

Reference

[1] Mostafa Y. Nassar, Hamed I. El-Salhy, W. H. El-Shiwiny, Gamal Abdelaziz, R. El-Shiekh, Composite Nanoarchitectonics of Magnetic Silicon Dioxide-Modified Chitosan for Doxorubicin Delivery and In Vitro Cytotoxicity Assay, *J. Inorg. Organomet. Polym. Mater.* **33** (2023) 237. Doi: https://doi.org/10.1007/s10904-022-02498-4

[2] A. M. Demin, A. V. Vakhrushev, A. G. Pershina, M. S. Valova, Lina V. Efimova, A. A. Syomchina, M. A. Uimin, A. S. Minin, G. L. Levit, V. P. Krasnov, V. N. Charushin, Magnetic-Responsive Doxorubicin-Containing Materials Based on Fe_3O_4 Nanoparticles with a SiO_2/PEG Shell and Study ofTheir Effects on Cancer Cell Lines, *Int. J. Mol. Sci.* **23** (2022) 9093. Doi: https://doi.org/10.3390/ijms23169093

[3] F. Zaaeri, M. Khoobi, M. Rouini, H. Akbari Javar, pH-responsive polymer in a core–shell magnetic structure as an efficient carrier for delivery of doxorubicin to tumor cells, *Int. J. Polym. Mater.* **67** (2018) 967. Doi: https://doi.org/10.1080/00914037.2017.1405348

[4] Z. Ferjaoui et al., Doxorubicin-Loaded Thermoresponsive Superparamagnetic Nanocarriers for Controlled Drug Delivery and Magnetic Hyperthermia Applications, *ACS Appl. Mater. Interfaces* **11** (2019) 30610. Doi: https://doi.org/10.1021/acsami.9b10444.

[5] Jorge L. Sanchez-Orozco, Luis A. García-Cerda, B. Puente-Urbina, H. Ivan Melendez-Ortiz, Poly(N-vinylcaprolactam-co-2-(diethylamino)ethylmethacrylate)coated Fe_3O_4@SiO_2 core-shell magnetic nanoparticles for controlled doxorubicin delivery, *J. Drug Deliv. Sci. Technol.* **81** (2023) 104253. Doi: https://doi.org/10.1016/j.jddst.2023.104253

[6] Q. Zhao, P. Xie, X. Li, Y. Wang, Y. Zhang, S. Wang, Magnetic mesoporous silica nanoparticles mediated redox and pH dual-responsive target drug delivery for combined magnetothermal therapy and chemotherapy, *Colloids Surf. A Physicochem. Eng. Asp.* **648** (2022) 129359. Doi: https://doi.org/10.1016/j.colsurfa.2022.129359

[7] Vy Anh Tran, Thu Thao Thi Vo, Sang-Wha Lee, Ngọc Don Tạ, Vo Vien, Van Dat Doan, Nguyen Chi Thanh, Van Thuan Le, Modulating drug retention and release via surface anchors using

hyperthermia and photothermal effects of Fe_3O_4-SiO_2 core-shell mesoporous nanoparticles, *J. Drug Deliv. Sci. Technol.* **102** (2024) 106396. Doi: https://doi.org/10.1016/j.jddst.2024.106396
